# A Novel Architecture Based on a Pyrrole-Functionalized Dithieno[3,2-b:2′,3′-d]pyrrole (DTP)-Type Conducting Polymer and Thiol-Modified Calixarene Derivative for Biophotovoltaic Solar Cells: Photocurrent and Hydrogen Generations via Both Photosynthesis and Respiratory System

**DOI:** 10.1021/acsomega.5c00164

**Published:** 2025-05-23

**Authors:** Mustafa Buyukharman, Huseyin Bekir Yildiz, Sumeyye Bakim, Mustafa Esen Marti

**Affiliations:** † Department of Physics, Institute of Graduate Studies in Science, 37516 Istanbul University, Istanbul 34134, Turkey; ‡ Department of Electrical-Electronics Engineering, Faculty of Engineering and Natural Sciences, 218507 KTO Karatay University, Konya 42020, Turkey; § Department of Computer Engineering, Faculty of Engineering and Natural Sciences, KTO Karatay University, Konya 42020, Turkey; ∥ Department of Chemical Engineering, Faculty of Engineering and Natural Sciences, 531804 Konya Technical University, Konya 42250, Turkey

## Abstract

This review explores the potential of biophotovoltaic devices (BPVs) as a sustainable solution for addressing the global energy crisis and combating climate change. BPVs generate renewable electricity from sunlight and water through the photosynthetic activity of microorganisms such as cyanobacteria and algae, which act as living photocatalysts. The study essentially focuses on improving photocurrent outputs through developing efficient anode materials. An innovative photoanode design is introduced employing cyanobacteria immobilized on a P­(DTP-Ph-Pyr)/Calixarene-AuNP-modified surface. This design features a porous structure conducive to cyanobacterial attachment and efficient electron transfer. As a first step, the conductive polymeric film of 4-(4-(1H-pyrrol-1-yl)­phenyl)-4H-dithieno­[3,2-b:2′,3′-*d*]­pyrrole (DTP-Ph-Pyr) monomer was coated onto a gold electrode via electropolymerization method. Then, a mixture of thiol- and carboxylic group-modified calixarene and gold nanoparticles (AuNPs) was applied to enhance the photoelectrode’s performance. The surface of the modified electrode enabled the successful immobilization of *Leptolyngbya* sp. cyanobacterial cells, providing a reliable interface for efficient photocurrent and hydrogen generation. Calixarenes and their derivatives act as favorable agents for cyanobacterial immobilization due to their specific configurations. Moreover, the formation of covalent bonds between the carboxyl groups of calixarenes and the amino groups in cyanobacteria facilitates the robust immobilization of cyanobacterial cells while maintaining their well-ordered structural integrity and organized cellular architecture. A complementary cathode structure, employing aniline-modified Pt nanoparticles, facilitates the reduction of protons to generate hydrogen gas. Overall, this study underscores the promise of BPVs as feasible clean energy technologies and introduces innovative methods to improve their efficiency and sustainability.

## Graphical abstract

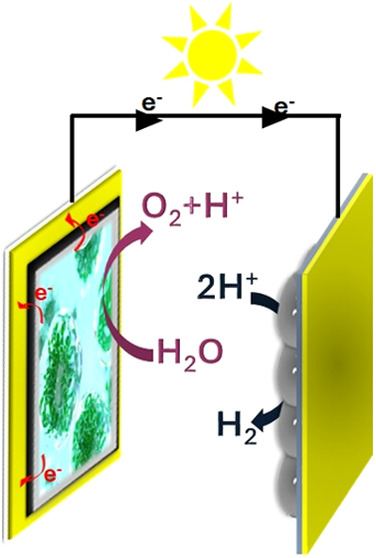

## 1 Introduction

Many nations are currently stepping up their efforts to replace fossil fuels with clean, sustainable, and innovative energy sources. Renewable energy resources are essential in formulating global policies for a sustainable future.^1^ In contrast to fossil fuels, which are limited and progressively unsustainable, researchers are concentrating on creating eco-friendly and economically viable energy alternatives. Research indicates that solar energy is the leading renewable resource, with solar energy conversion systems exhibiting superior efficiency and sustainability relative to other options.^2^ Biophotovoltaic solar cells (BPVs) are innovative systems that exploit the photosynthetic activity of microorganisms, such as algae or cyanobacteria, to generate renewable electricity. These systems integrate living biological entities with conductive electrodes to convert solar energy into electrical energy through light-driven biochemical reactions.^3^ While traditional microbial fuel cells require an external organic substrate to act as an electron donor, BPV systems depend on solar light to split water and supply the resulting electrons to the system through the photosynthetic capability of microorganisms.^4^ Compared to traditional photovoltaic (PV) technology, biophotovoltaics (BPV) are regarded as more environmentally sustainable due to their use of nontoxic and renewable photosynthetic materials. Unlike conventional PV systems that generate electricity only during daylight, biophotovoltaic (BPV) systems have the capability to sustain electricity generation under dark conditions through the oxidation of intracellular metabolites, which serve as endogenous electron donors in the absence of light.^5^ Additionally, BPVs exhibit intrinsic energy storage capabilities, operating analogously to rechargeable batteries by undergoing separate charge and discharge phases. This inherent storage function sets BPV systems apart from conventional photovoltaic technologies, which lack the capacity for direct energy storage.^6^ In addition to energy generation and storage, BPVs can serve as multifunctional platforms, such as biosensors for detecting environmental pollutants, further expanding their applicability in environmental monitoring.^7^ In addition, photosynthetic microorganisms used in BPVs can be cultured at a low cost. As a result, owing to these compelling advantages, BPVs have emerged as a focal point of growing scientific interest.

Cyanobacteria stand out among photosynthetic organisms as preferred candidates for power generation in biophotovoltaic (BPV) systems, owing to their remarkable energy conversion efficiency, ability to produce electricity directly, and inherent environmental sustainability.^8^ In recent years, a growing body of research has explored cyanobacteria-based biophotovoltaic (BPV) systems, employing strains such as *Geobacter sulfurreducens*,^9^ *Synechococcus* sp. PCC 7942, Anabaena variabilis,^10^ *Synechocystis* sp. PCC 6803,^11,12^ and *Leptolyngbya* sp.,^7,13,14^ immobilized on various electrode materials including graphite, carbon brushes, platinum, gold, and indium tin oxide (ITO) glass. These systems aimed to achieve enhanced photocurrent generation, ranging from 500 nA to 400 μA, by facilitating efficient electron transfer pathways. Howe and colleagues demonstrated the capability of cyanobacteria-based BPVs to generate electricity in both illuminated and dark conditions without the use of mediators, achieved through the development of biofilms composed of cyanobacteria.^8^ Several studies have demonstrated that the prolonged retention of cyanobacteria within BPV devices significantly contributes to improved and sustained power output performance over time.

The term “calixarene” originates from the structural resemblance of these molecules—particularly in their space-filling representation—to the calix crater, an ancient Greek vessel, highlighting the vase-like architecture of the macrocyclic framework.^15^ Calixarenes represent a group of cyclic oligomeric compounds typically obtained via the acid- or base-catalyzed condensation of phenol with formaldehyde. Due to their straightforward synthesis, vast potential for functionalization, and ability to form derivatives with varying cavity sizes, calixarenes have emerged as highly versatile organic compounds. They have been extensively employed across diverse disciplines, serving roles as selective carriers in enzyme mimicry, solid-phase supports, ion transport agents, drug delivery platforms, ion-selective electrodes, chemical and biological sensors, as well as in various catalytic applications.^16^ Recent studies have highlighted the incorporation of calixarenes in photocurrent generation studies, particularly in enhancing the efficiency of solar cells. Acting as molecular scaffolds, calixarenes play a pivotal role in facilitating charge separation and electron transfer, improving light-harvesting capabilities, and enhancing the overall efficiency of solar energy technologies. Additionally, the unique cavity structure of calixarenes enables effective deposition of guest molecules onto surfaces, underscoring their potential as exceptional host macrocyclic molecules. This distinct feature positions calixarenes as promising candidates for the development of innovative BPV systems.^17–20^

Dithieno­[3,2-b:2′,3′-d]­pyrrole (DTP)-based conductive polymers exhibit several advantageous properties that establish them as promising candidates for biophotovoltaic (BPV) applications. The planar and electron-rich architecture of the DTP moiety facilitates efficient π–π stacking interactions, which significantly enhance charge carrier mobility—a critical factor for the efficient operation of BPV devices requiring rapid and reliable charge transport.^21^ Additionally, DTP-based polymers display strong light absorption across a broad spectrum of solar radiation, thereby improving the light-harvesting efficiency of BPV systems and optimizing the utilization of incident sunlight.^22^ The chemical structure of DTP offers exceptional versatility as it allows for functionalization at various positions. This enables the synthesis of polymers with precisely tailored electronic and optical properties to meet the specific demands of BPV systems.^23,24^ Moreover, these polymers exhibit excellent thermal stability and resistance to environmental degradation, ensuring the durability and prolonged operational lifespan of BPV devices.^25^ Collectively, these characteristics underscore the potential of DTP-based conductive polymers to enhance the performance and stability of BPV systems, contributing to advancements in efficient energy conversion technologies.

Hydrogen can currently be produced through various methods, including fossil fuel reforming, conversion of natural gas and biobased liquids, gasification of coal and biomass, thermochemical and nuclear-based processes, as well as water decomposition via electrolysis or photoelectrochemical methods.^26–31^ However, these technologies are often limited by high costs or environmental concerns. While a range of chemical techniques exist for hydrogen production, microbial biotechnologies have garnered significant attention due to their renewable nature and cost-effectiveness, particularly in the context of sustainability. Under anaerobic conditions, microbial cells can ferment liquid suspensions with a high carbohydrate content, such as those found in industrial and household wastewater, to produce hydrogen. As an alternative, microorganisms performing photosynthesis can facilitate photoinduced biological hydrogen generation, using water and sunlight as abundant and renewable resources without the need for organic matter supplementation. Despite these promising capabilities, research involving biophotovoltaic (BPV) systems for hydrogen production remains limited, particularly in systems employing the anode surface functionalized with microorganisms that can perform photosynthesis, such as cyanobacteria, to generate hydrogen at the cathode. Recent studies have demonstrated the potential of BPVs incorporating microbial species capable of performing photosynthesis to generate a photoelectrical current and hydrogen gas. For instance, Howe and colleagues employed *Synechocystis* sp. in a cyanobacteria-based BPV, utilizing [Fe­(CN)_6_]^4–^/^3–^ as an electron mediator to generate photocurrent under both light and dark conditions. During illumination, protons produced via photosynthesis facilitated hydrogen generation in the dark environment, with an applied bias potential of 1–1.4 V.^32^ Similarly, Saper et al. investigated *Synechocystis* sp. in cyanobacteria-based BPVs, achieving photocurrent and hydrogen production through photosynthetic activity. By introduction of the PS II inhibitor 3-(3,4-dichlorophenyl)-1,1-dimethylurea (DCMU), which inhibits the activity of photosystem II (PS II) responsible for water splitting and oxygen evolution in photosynthesis, the cyanobacteria were redirected toward the respiratory pathway, generating electrons and protons via photosystem I (PS I), which facilitates electron transfer for ATP and NADPH production. Their results showed enhanced hydrogen and photocurrent generation when exogenous glucose was introduced, as the respiratory system initially oxidized the supplied carbon substrates.^33^ Furthermore, significant photocurrent and hydrogen production were demonstrated via electrospun graphene-cellulose acetate/cyanobacteria^34^-based BPV. These findings underscore the potential of BPVs for enhancing both photocurrent and hydrogen generation, advancing the development of sustainable energy systems and achieving substantial improvements in energy conversion efficiency.

This study presents an innovative photoanode design that utilizes cyanobacteria immobilized on a structure of novel pyrrole-functionalized DTP polymer (P­(DTP-Ph-Pyr)) and calixarene derivative with AuNPs (P­(DTP-Ph-Pyr)/calixarene-AuNP) to generate not only photocurrent and hydrogen photosynthetically but also to harness the respiratory system of cyanobacteria for production of hydrogen and photocurrent. The polymer film was synthesized through the direct electropolymerization of the pyrrole-functionalized monomer, namely, 4-(4-(1H-pyrrol-1-yl)­phenyl)-4H-dithieno­[3,2-b:2′,3′-d]­pyrrole (DTP-Ph-Pyr). Subsequently, a mixture comprising calixarene and AuNPs was employed to functionalize the surface coated with the conducting polymer for creating an improved platform for cyanobacteria immobilization. The modified surface was biofunctionalized by immobilizing *Leptolyngbia* sp. cyanobacteria through covalent bond coupling compounds, which are *N*-hydroxysuccinimide (NHS) and N-(3-(dimethylamino)­propyl)-N-ethylcarbodiimide hydrochloride (EDC). Functional groups bearing carboxylic acids at the top rim of the calixarene structure enabled the covalent capture of cyanobacterial cells with high specificity. The establishment of covalent bonds between cyanobacteria and calixarene constitutes the most robust attachment method. The porous structure of the P­(DTP-Ph-Pyr)/Calixarene/AuNP composite enhances efficient electron transfer by offering optimal accommodation sites for cyanobacteria and minimizing internal resistance within the BPV system. By this motivation, the conductive polymer P­(DTP-Ph-Pyr) was employed to enhance the cumulative effectiveness of the BPV by facilitating more efficient charge transfer, thereby promoting increased photocurrent generation and hydrogen production. Calixarenes are macrocyclic molecules with a bowl-shaped amphiphilic architecture, in which a water-attracting outer region and a hydrophobic cavity support enhanced affinity toward biological species.^35^ Their unique hydrophilic and hydrophobic properties allow calixarenes to bind selectively with a broad variety of guest molecules. These features facilitate efficient cyanobacteria loading and provide an ideal site for selective binding with guest molecules, such as cyanobacteria, through covalent attachment. In addition, gold nanoparticles (AuNPs) were utilized to enable selective biomolecular interactions, owing to their extensive surface area, exceptional compatibility with biological systems, and outstanding electrical conductivity, which supports rapid electron transfer.^7,35,36^ Thiol moieties function as specific binding domains that allow calixarenes to be covalently immobilized on gold nanoparticles through gold–sulfur interactions. Considering the significance of the conducting polymer, calixarene, and AuNPs, the combined effects of these materials on the performance of the BPV were evaluated. Additionally, the cathode of the BPV was fabricated by electrochemical binding of aniline-modified Pt nanoparticles to conductive aniline-functionalized DTP polymer, namely, poly­(4-(4H-dithiopheno­[3,2-b:2′,3′-d]­pyrol-4-yl)­aniline), P­(DTP-Ph-NH_2_)-coated gold electrode, via conductive oligoaniline bridges.^37^ Conductivity of P­(DTP-Ph-NH_2_) and the oligoaniline bridges augmented the efficiency of hydrogen production. Under applied constant potential and visible light (white light) illumination, when the system was operated, water was oxidized by cyanobacteria through photosynthetic activity, resulting in electron release, which in turn generated photocurrent upon electron transport from the cyanobacteria surface to the (anode) electrode. At the same time, PtNPs on the cathode surface facilitated the reduction of protons, leading to the evolution of hydrogen gas. When the PS II inhibitor DCMU was introduced into the medium to inhibit the photosynthesis activity of cyanobacteria and the system was illuminated under constant potential in a glucose-rich environment, cyanobacteria started to use their respiratory property and electrons produced by glucose oxidation in photosystem I (PS I) in cyanobacteria were transferred to the anode. This process enabled the simultaneous generation of photocurrent and hydrogen gas through the transport of electrons and proton reduction at the cathode.

In recent years, BPV research has attracted significant attention. Research has indicated that BPVs based on photosynthetic microorganisms possess the ability to produce both hydrogen and photoelectrical current. The majority of recent BPV studies predominantly target the generation of electricity and/or hydrogen gas. In this study, the BPV system harnesses both the photosynthesis and respiration capabilities of cyanobacteria to produce photocurrent and hydrogen. Current research addressing energy conversion processes in biophotovoltaic (BPV) systems—particularly those leveraging photosynthetic organisms and cellular respiration pathways for simultaneous electricity and hydrogen production, remains scarce and is just about absent in the existing literature. This study highlights the potential for electricity and hydrogen generation under conditions where photosynthesis is limited by leveraging the respiration feature of cyanobacteria.

## 2 Experimental Section

### 2.1 Materials

Dichloromethane (DCM), acetonitrile (ACN), tetrabutylammonium hexafluorophosphate (TBAPF_6_), and dimethylformamide (DMF) were obtained from Merck (Darmstadt, Germany). *N*-Hydroxysuccinimide (NHS) and *N*-(3-(dimethylamino)­propyl)-N-ethylcarbodiimide hydrochloride (EDC) were purchased from Fluka (Buchs, Switzerland) and Sigma, respectively. Gold nanoparticles (10 nm diameter), stabilized in 0.1 mM phosphate-buffered saline (PBS), were supplied by Sigma-Aldrich. All additional chemicals required for the synthesis of the monomer were also procured from Sigma-Aldrich and used without further purification. *Leptolyngbya* sp., a filamentous cyanobacterium widely recognized among photosynthetic microorganisms, was obtained from Carolina Biological Supply. The strain was cultivated under standard growth conditions in accordance with established procedures previously reported in the literature.^7,13^ A Modified Leonian Agar (MLA) complex solution, recognized as an appropriate growth medium for cyanobacteria, was utilized. The incubation process was conducted at ambient temperature under conditions of low ionic strength, with illumination provided by a white, fluorescent lamp emitting photons at a power of 40 μmol, set to a 12:12 (hours) light/dark cycle. After incubation, the cells were subjected to centrifugation at 20 °C for 10 min at 4000 rpm, followed by washing with MLA solution and a subsequent round of centrifugation under identical conditions. The harvested *Leptolyngbya* sp. cells were resuspended in MLA medium at a concentration of 1 g/mL and immediately utilized for photoelectrochemical measurements. All additional reagents were procured from Sigma-Aldrich and employed without further purification. Ultrapure water obtained from a Nanopure purification system (Barnstead) was used in all of the experimental procedures.

### 2.2 Methods

#### 2.2.1 The Design of the Photoanode Electrode

##### 2.2.1.1 Synthesis of 4-(4-(1H-Pyrrol-1-yl)­phenyl)-4H-dithieno­[3,2-b:2′,3′-d]­pyrrole

The monomer 4-(4-(1H-pyrrol-1-yl)­phenyl)-4H-dithieno­[3,2-b:2′,3′-d]­pyrrole (denoted as DTP-Ph-Pyr) was synthesized following the procedure described in a previous study.^38^ The synthetic route involved the coupling reaction of 3,3′-dibromo-2,2′-bithiophene with 4-(1H-pyrrol-1-yl)­aniline in the presence of BINAP as the catalyst and sodium tert-butoxide (NaOtBu) as the base. The reaction was conducted at a temperature of 110 °C for a duration of 12 h, resulting in the successful formation of the target monomer, DTP-Ph-Pyr, as outlined in Scheme 1A. The detailed information about the synthesis and characterization of DTP-Ph-Pyr monomer and electrochemical polymerization and characterization of its polymer P­(DTP-Ph-Pyr) (including Figure S1) is given in the Supporting Information.

**Scheme 1:**
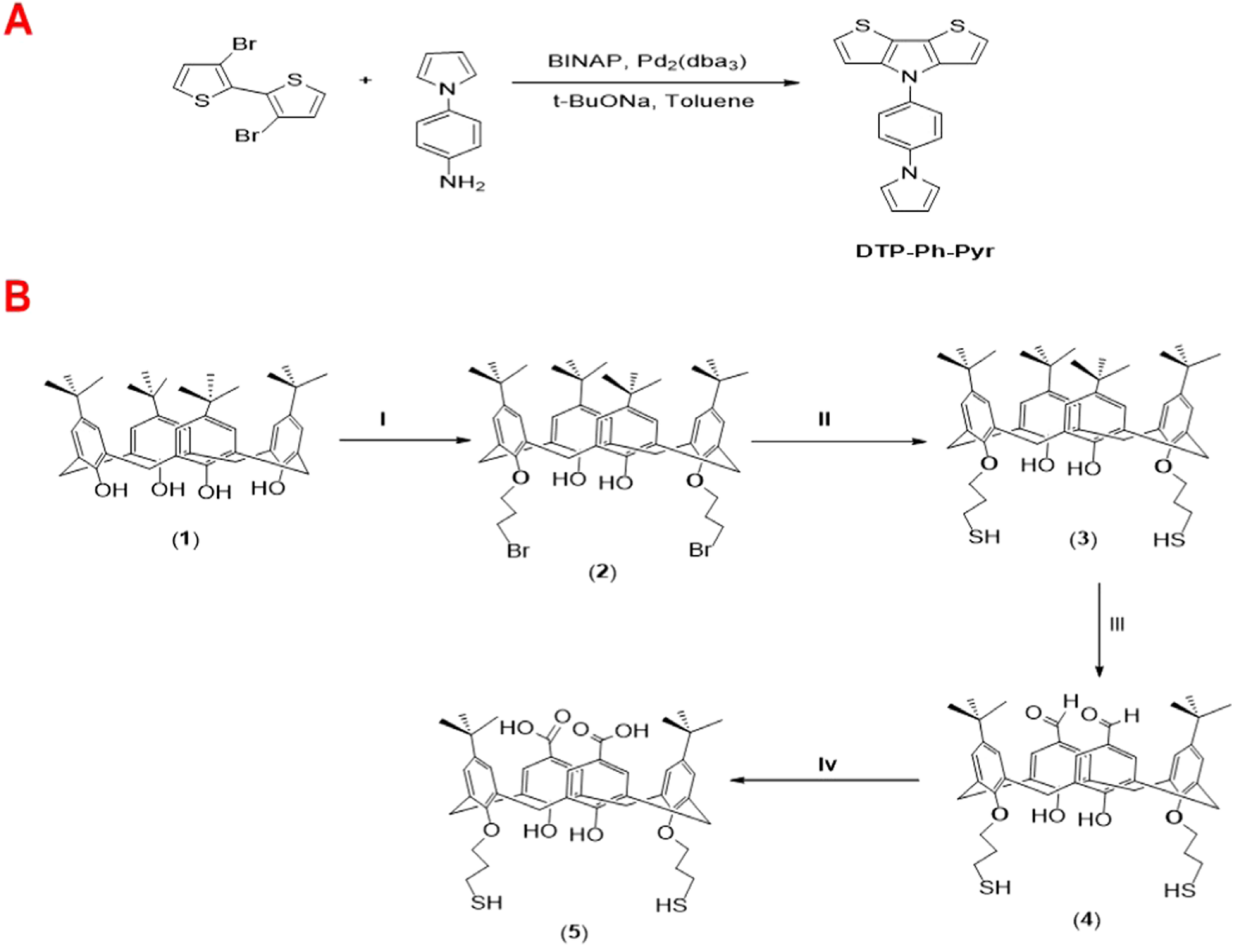
Synthetic Routes for (A) 4-(4-(1H-pyrrol-1-yl)­phenyl)-4H-dithieno­[3,2-b:2′,3′-d]­pyrrole (DTP-Ph-Pyr) monomer; (B) 5,17-Bis-*tert*-butyl-11,23-dicarboxylic acid-25,27-dihydroxy-26,28-bis­(3-thiol-1-oxypropane)-calix [4]­arene (5)^a^

a Reaction conditions: (i) K_2_CO_3_, CH_3_CN, 1,3-dibromopropane; (ii) thiourea, KOH, CH_3_CN; (iii) TFA, hexamethylenetetramine; (iv) sulfamic acid, sodium chlorite, CHCl3/acetone; Calixarene labeld as (1): 5,11,17,23-tetra-tert-buthyl-25,26,27,28-hydroxycalix [4]­arene; Calixarene labeld as 2:5,11,17,23-tetra-*tert*-butyl-25,27-bis­(3-bromo-1-oxypropane)-calix [4]­arene; Calixarene labeled as (3): 5,11,17,23-tetra-*tert*-butyl-25,27-dihydroxy-26,28-(3-thiol-1-oxypropane)-calix [4]­arene and Calixarene labeşed as (4): 5,17-Bis-*tert*-butyl-11,23-dicarboxaldehyde-25,27-dihydroxy-26,28-bis­(3-thiol-1-oxypropane)-calix [4]­arene.

##### 2.2.1.2 Synthesis of the Calixarene

The synthesis steps for the calixarene compound utilized in this study, identified as 5,17-bis-tert-butyl-11,23-dicarboxylic acid-25,27-dihydroxy-26,28-bis­(3-thiol-1-oxypropane)-calix[4]­arene (labeled as 5), are illustrated in Scheme 1B. The intermediate calixarene compounds 5,11,17,23-tetra-*tert*-buthyl-25,26,27,28-hydroxycalix [4]­arene (labeled as 1), 5,11,17,23-tetra-*tert*-butyl-25,27-bis­(3-bromo-1-oxypropane)-calix [4]­arene (labeled as 2), and 5,11,17,23-tetra-*tert*-butyl-25,27-dihydroxy-26,28-(3-thiol-1-oxypropane)-calix [4]­arene (labeled as 3) were prepared following procedures published in the literature.^36,39^ The synthesis of 5,17-Bis-*tert*-butyl-11,23-dicarboxaldehyde-25,27-dihydroxy-26,28-bis­(3-thiol-1-oxypropane)-calix [4]­arene (labeled as 4) and the final product, 5,17-bis-tert-butyl-11,23-dicarboxylic acid-25,27-dihydroxy-26,28-bis­(3-thiol-1-oxypropane)-calix [4]­arene (labeled as 5), were carried out in accordance with methods in the previously reported the study^40^ (Scheme 1B).

##### 2.2.1.3 Fabrication Process of the Photoanode

The desired monomer, DTP-Ph-Pyr, was first electrochemically polymerized onto a precleaned bare gold electrode. The electropolymerization of DTP-Ph-Pyr was performed via cyclic voltammetry (*C–V*) by repetitively cycling the potential swept between −0.5 and +1.5 V at a scan rate of 100 mV s^–1^. The polymerization was conducted in an electrolyte solution composed of 0.1 M TBAPF_6_ dissolved in DCM.^38^ During this process, a graphite rod electrode (diameter: 5 mm) was employed as the counter electrode, while a Ag/AgCl electrode served as the reference. Upon completion of polymerization, the electrode surface was thoroughly rinsed with distilled water to eliminate residual impurities. Separately, an appropriate amount of calixarene was dissolved in 0.1 mL of dimethylformamide (DMF) within an Eppendorf tube, and the mixture was stirred at room temperature to ensure complete dissolution. A calixarene–gold nanoparticle (Calixarene/AuNP) suspension was subsequently prepared by mixing the calixarene solution (5 mg/mL) with AuNPs in a 1:9 (v/v) ratio. The resulting suspension was drop-cast onto a polymer-coated electrode surface. The P­(DTP-Ph-Pyr)-coated electrode was then left at room temperature for 24 h to enable covalent attachment between the thiol groups of calixarene and the AuNPs. Following the coating of bare gold electrodes with the P­(DTP-Ph-Pyr)/Calixarene/AuNP structure, cyanobacteria were immobilized on the modified electrode surface. This was achieved by applying the cyanobacteria dropwise using N-(3-(dimethylamino)­propyl)-N-ethylcarbodiimide hydrochloride (EDC) and *N*-hydroxysuccinimide (NHS) as cross-linking agents to make a covalent bond. These coupling reagents were utilized to functionalize the free carboxylic acid moieties present on the calixarene framework.^41^ Following the modification step, the electrode was left to dry at ambient temperature for approximately 3 h (Scheme 2).

**Scheme 2:**
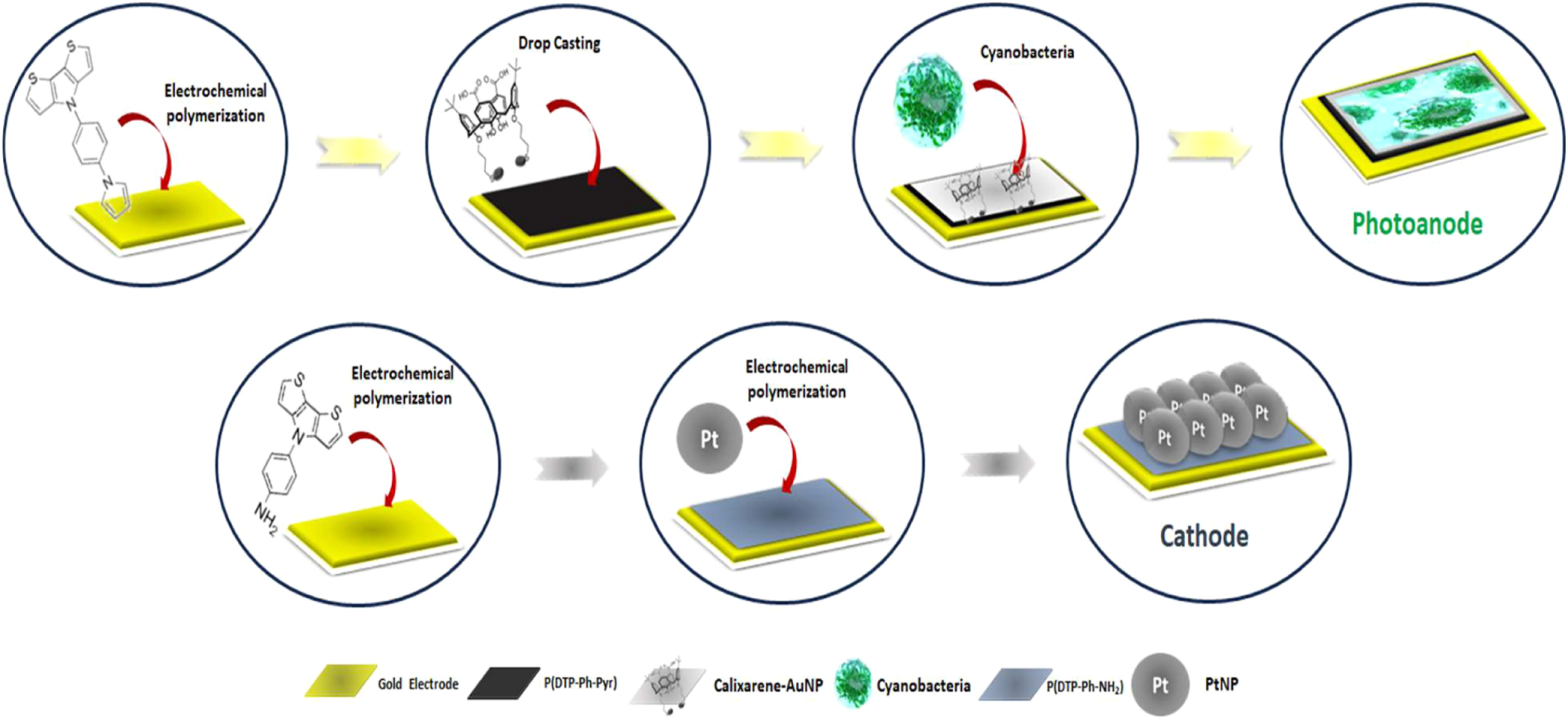
Schematic Representation for the Preparations of the Photoanode and Cathode of the P­(DTP-Ph-Pyr)/Calixarene-AuNP/Cyanobacteria-Based BPV

#### 2.2.2 The Construction of Cathode Electrode

The synthesis of the monomer 4-(4H-dithiopheno­[3,2-b:2′,3′-d]­pyrol-4-yl)­aniline (DTP–Ph–NH_2_), along with its electrochemically produced homopolymer, was conducted in accordance with previously reported methodologies^42^ (The detail information about the synthesis and characterization of DTP-Ph-NH_2_ monomer and electrochemical polymerization and characterization of its polymer P­(DTP-Ph-NH_2_) (including Scheme S1 and Figure S2) is given in the Supporting Information). Additionally, aniline-functionalized platinum nanoparticles (PtNPs) were prepared following established protocols available in the literature^43^ (The detailed information about the synthesis and characterization of AuNPs (including Figure S3) is given in the Supporting Information). The DTP–Ph–NH_2_ monomer, at a concentration of 3 × 10^–2^ M, underwent electropolymerization using the cyclic voltammetry (*C–V*) method in a 0.1 M TBAPF_6_/DCM/ACN medium with a scan rate of 100 mV/s. The fabrication of the cathode electrode was completed through electropolymerization of the DTP–Ph–NH_2_ homopolymer and aniline-modified PtNPs in a 0.1 M phosphate buffer solution (pH 7.4). This process was performed via *C–V* within a potential range of −0.1 V to +1.1 V at a scan rate of 100 mV/s, where the components were interconnected through oligoaniline bonds The detailed information about the electrochemical characterization of oligoaniline bridges between P­(DTP-Ph-NH_2_) and aniline-modified PtNPs (including Figure S4) is given in the Supporting Information. During these experiments, a graphite rod electrode (diameter: 5 mm) was utilized as the counter electrode, while an Ag/AgCl electrode functioned as the reference. The experimental setup is illustrated in Scheme 2.

#### 2.2.3 Integration of the BPV Cell

In the photocurrent experiments, H-type cells were employed, with an Au electrode modified with P­(DTP–Ph–Pyr)/Calixarene/AuNP/Cyanobacteria serving as the anode in the biophotovoltaic (BPV) cell and another Au electrode coated with P­(DTP–Ph–NH_2_)/PtNP, positioned at a distance of 50 mm, functioning as the biocathode. These electrodes were placed in separate tubular compartments facing each other, separated by a proton-permeable Nafion membrane at the same 50 mm distance. A multimeter was connected across an external resistor to monitor the output. Photocurrent measurements were carried out using a solar simulator integrated with a custom-built photochemical system. The photoelectrochemical setup consisted of an Oriel 300 W xenon lamp (Model 6258), an Oriel monochromator with a spectral resolution of 2 nm (Model 74,000), and an Oriel optical chopper (Model 76,994). Signal acquisition and digital processing were carried out using a phase-sensitive lock-in amplifier (Stanford Research Systems, Model SR830 DSP). The cutoff frequency of the current signal was modulated using a pulse-retardant generator (Stanford Research Systems). For measurements conducted under constant potential conditions, a potentiostat/galvanostat (EG&G Model 263) was employed in conjunction with the aforementioned electrode configuration to ensure precise electrochemical control and reproducibility.

Scheme 3 depicts the photocurrent and hydrogen generation processes within the BPV system. Under constant applied potential and visible light (white light) illumination, the system facilitated water splitting through oxidation, driven by photosynthesis in cyanobacteria. This process generated photocurrent, as electrons, released from water oxidation, were transferred to the anode. Simultaneously, hydrogen gas was produced on the cathode side through the electrochemical reduction of protons via the PtNPs.

**Scheme 3:**
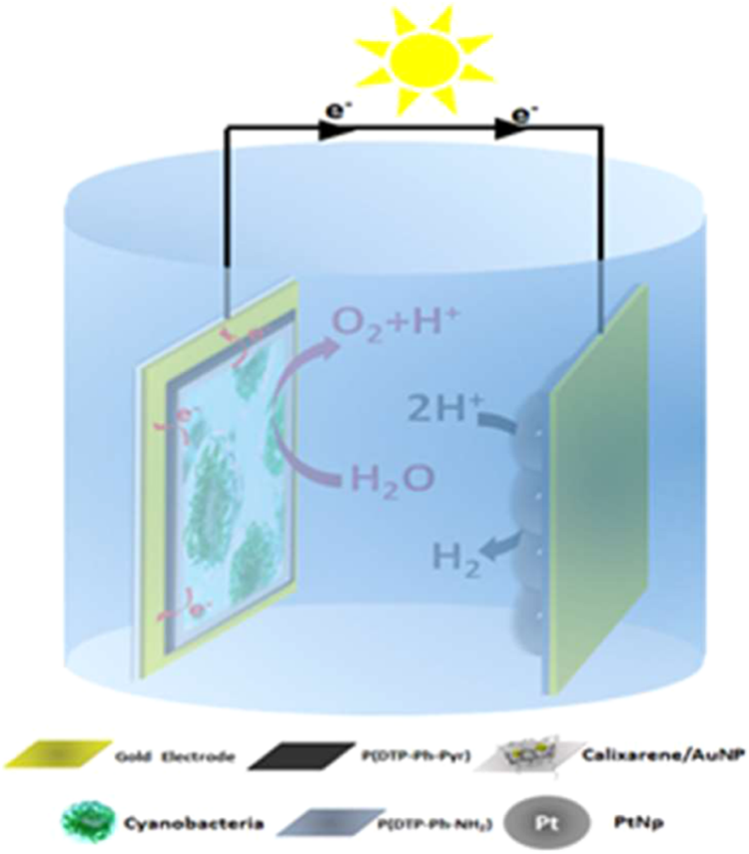
Schematic Representation of Working Principle, Electron Transfer Mechanism, and Integration of the P­(DTP-Ph-Pyr)/Calixarene-AuNP/Cyanobacteria-Based BPV

The hydrogen generation process within the BPV system was carried out under varying illumination durations (2.5, 5, 10, 15, 20, 25, and 30 min) at a light intensity of 1400 W/m^2^ (Scheme 3). For the analysis of hydrogen gas, a 1 mL (cm^3^) gas sample was withdrawn from the system using a gastight syringe and subsequently analyzed by gas chromatography (Agilent 6890N).

## 3 Results and Discussion

### 3.1 The Optimization Studies for Cathode (P­(DTP-Ph-NH_2_)/PtNP) Electrode of the BPV Cell

A key characteristic of platinum nanoparticles (PtNPs) lies in their ability to catalyze the electrochemical reduction of hydrogen peroxide (H_2_O_2_) to water. The real purpose of the study is that PtNPs would reduce the protons produced by the oxidation of water via photosynthesis done by cyanobacteria to hydrogen gas. It was thought that surface optimization parameters enabling the most rapid electron transfer from the electrode to the PtNPs could be determined through the electrochemical reduction of hydrogen peroxide. Furthermore, it was assumed that the optimal conditions derived from this process would also be applicable to facilitate efficient proton reduction and subsequent hydrogen gas evolution during water splitting reaction via photosynthesis in the BPV cell.^31,43^ The first characterization study of the P­(DTP-Ph-NH_2_)/PtNP electrode, which served as the cathode in our water separation system, involved determining the optimum number of cycles needed to connect Pt nanoparticles to a 1 cm^2^ P­(DTP-Ph-NH_2_)-modified gold electrode via oligoaniline bonds. In this optimization study, Pt nanoparticles (PtNPs) were electropolymerized onto a P­(DTP-Ph-NH_2_) conductive polymer film, which had been previously synthesized through 100 cyclic voltammetry scans. Throughout the process, the PtNP concentration was maintained at a constant level of 1 mg/mL. All electrochemical experiments were performed in a phosphate-buffered solution at pH 7.4. During this optimization study, PtNPs were attached to the electrode covered with the P­(DTP-Ph-NH_2_) conductive polymer by using oligoaniline composite bonds via electropolymerization at cycle numbers of 40, 60, 80, and 100. To find this optimum, P­(SNS-Ph-NH_2_)/PtNP electrodes were tested under potential differences ranging from 1.0 V to −0.5 V in a medium containing 9 mM H_2_O_2_. The results, obtained from the net current differences in the voltammograms due to the reduction of H_2_O_2_ to water by PtNPs, revealed that the optimum cycle number required for binding PtNPs to the conductive polymer was 80. It was observed that increasing the number of electropolymerization cycles during composite formation led to a progressive enhancement in the current response recorded in the voltammograms, with the maximum difference attained at 80 cycles. However, beyond 80 cycles, the current difference began to decrease (Figure S4a). This decrease could be attributed to the rapid increase in Pt nanoparticles as the number of cycles increased, leading to congestion in the conductivity paths and ultimately reducing the efficiency of electron transfer. This optimization experiment, repeated three times with consistent results, concluded that 80 cycles were optimal for binding Pt nanoparticles to the P­(DTP-Ph-NH_2_) polymer with composite oligoaniline bonds (Figure S5A).

In an additional study focusing on optimization, we aimed to determine the optimum cycle number for electropolymerizing the DTP-Ph-NH_2_ monomer using cyclic voltammetry. The P­(DTP-Ph-NH_2_)/PtNP-modified gold electrode, intended for use as the cathode in our project, was obtained by applying Pt nanoparticles at a fixed concentration of 1 mg/mL on a polymeric layer prepared by using different numbers of electropolymerization cycles. Pt nanoparticles were applied via electropolymerization using 80 cycles in alternating voltammetry. Cyclic voltammetry measurements were subsequently performed over a potential window ranging from 0.2 to −0.5 V in a phosphate buffer solution (pH 7.4) containing 9 mM H_2_O_2_. The results showed that the maximum current variation observed in the voltammograms obtained via cyclic voltammetry was observed with the P­(DTP-Ph-NH_2_) polymer electropolymerized for 100 cycles. However, when the polymer electrode was electropolymerized for 120 cycles, the current difference decreased. This decrease was ascribed to the attainment of an optimal polymer film thickness and conductivity at 100 electropolymerization cycles, beyond which further increases did not enhance the electron transfer efficiency. Beyond 100 cycles, the polymer film became too thick, hindering electron transfer and resulting in insufficient electrons reaching the electrode (Figure S5B). In this optimization study, P­(DTP-Ph-NH_2_)/PtNP-modified gold electrodes with an area of 1 cm^2^ were utilized, and the study was repeated three times, yielding consistent results each time.

### 3.2 Photoelectrochemical Performance of the Photoanode

A series of control experiments were carried out to elucidate the underlying mechanism of photocurrent generation by the gold electrode functionalized with the P­(DTP-Ph-Pyr)/Calixarene-AuNP/Cyanobacteria structure. Initially, cyanobacteria were immobilized onto the surface of a gold electrode, and after a while at room temperature to dry the electrode, it was placed in a phosphate-buffered medium (pH = 7.4). No photocurrent was observed in this setup when the 1400 W/m^2^ (1 sun unit) visible region light was sent to the system. In a subsequent experiment, the cyanobacteria-coated gold electrode produced a photocurrent of 23 μA upon immersion in a phosphate buffer solution supplemented with 0.5 μmol of phenyl-p-benzoquinone, which served as an electron transfer mediator (Figure 1A). These results suggest that a mediator is required to facilitate electron transfer generated by water oxidation during photosynthesis. All control experiments were carried out at a fixed applied potential of 0.0 V while maintaining the cyanobacterial concentration consistently at 750 mg/mL. In an additional control experiment, cyanobacteria at a concentration of 750 mg/mL were immobilized onto a gold electrode surface modified with a P­(DTP-Ph-Pyr) polymeric film, which had been fabricated through 40 cycles of electropolymerization. Upon immersion of the gold electrode modified with the P­(DTP-Ph-Pyr)/cyanobacteria assembly into the buffer solution and subsequent illumination, a substantial increase in photocurrent was observed, reaching 94 μA under a constant applied potential of 0 V (Figure 1B). This result demonstrates that the conductive polymeric film improved the electron transfer rate during photosynthesis because of having pyrrole moiety that enables stronger π–π stacking interactions, improved charge carrier mobility, and facilitates more efficient electron transport. During the immobilization process, when a photoanode composed solely of P­(DTP-Ph-Pyr) was employed, the hydrophobic nature of the polymer’s pendant alkyl chains hindered effective interaction with the cyanobacteria. As a result, stable immobilization on the polymer surface was challenging and a portion of the cyanobacterial cells detached and leached from the electrode interface. Therefore, to prevent leaching of cyanobacteria from the electrode surface, a dialysis membrane, stored in 0.1 M phosphate buffer solution at pH 7.4, was cut and positioned to cover the electrode surface and secured with an O-ring to fix cyanobacteria. Furthermore, the lack of AuNPs and calixarene hindered the appropriate spatial orientation of the biomolecule at the electrode interface, thereby compromising its effective immobilization. In the final control experiment, the conjugated polymer film was deposited onto the gold electrode after 40 cycles of electropolymerization. Calixarene-AuNP suspension containing 0.50 mg of calixarene was dropped onto the polymer film-coated electrode surface. Following the entrapment of cyanobacteria at a concentration of 750 mg/mL within the composite structure, the gold electrode modified with the P­(DTP-Ph-Pyr)/Calixarene-AuNP/Cyanobacteria assembly was immersed in the buffer solution and subsequently exposed to visible light irradiation at an intensity of 1400 W/m^2^. The constructed photoanode P­(DTP-Ph-Pyr)/Calixarene-AuNPs/Cyanobacteria showed outstanding features and gave an ideal platform for the most effective immobilization in electrically connected targets to generate a high photocurrent. Therefore, the photocurrent intensity exhibited a notable enhancement, reaching up to 371 μA (Figure 1C). In the final control setup, the gold electrode functionalized with the P­(DTP-Ph-Pyr)/Calixarene-AuNP/Cyanobacteria construct was immersed in absolute ethanol and subjected to visible light illumination. No photocurrent generation was detected. This finding highlights the structure’s water-specific sensitivity, indicating that photocurrent generation is governed by electron transfer processes that are critically dependent on water as the principal electron donor.

**Figure 1:**
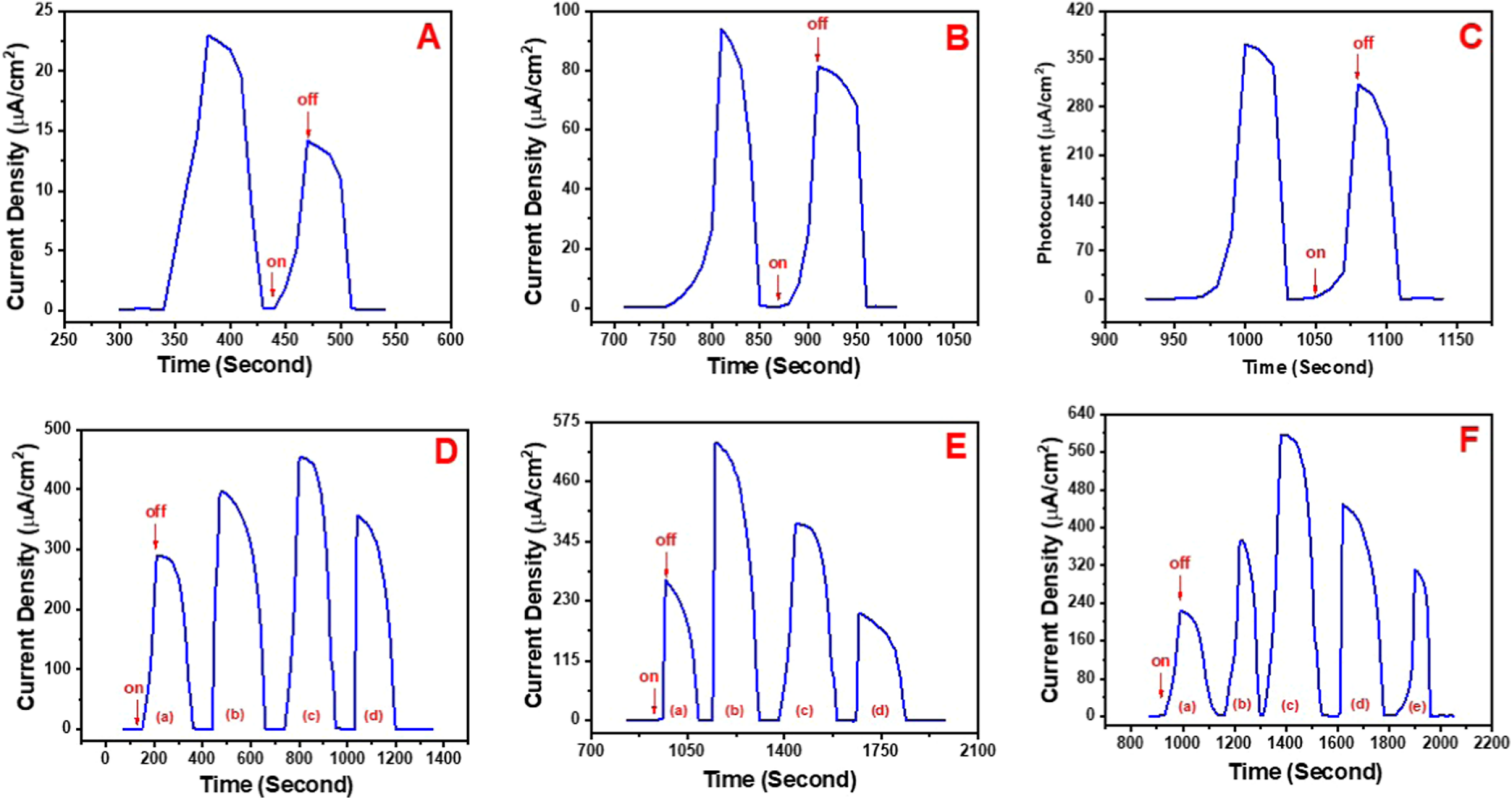
(A) Chronoamperometry studies of the bare Au electrode coated with 750 mg/mL cyanobacteria in the presence of 0.50 μmol of phenyl-p-benzoquinone. (B) Chronoamperometry studies of the gold electrode modified with the P­(DTP-Ph-Pyr) (40 cycles)/Cyanobacteria (750 mg/mL) structure. (C) Chronoamperometry studies of the gold electrode modified with the P­(DTP-Ph-Pyr) (40 cycles)/Calixarene-AuNP (0.5 mg of calixarene)/Cyanobacteria (750 mg/mL) structure. (D) Chronoamperometry studies of the P­(DTP-Ph-Pyr) (40 cycles)/Calixarene-AuNP/Cyanobacteria (750 mg/mL) photoanode structure including different amounts of the Calixarene (a) 0.25 mg, (b) 0.50 mg, (c) 0.75 mg and, (d) 1.0 mg. (E) Chronoamperometry studies of the P­(DTP-Ph-Pyr) (40 cycles)/Calixarene-AuNP (0.75 mg of calixarene)/Cyanobacteria photoanode structure with different cyanobacteria concentrations: (a) 250 mg/mL, (b) 500 mg/mL, (c) 750 mg/mL, and (d) 1000 mg/mL. (F) Chronoamperometry studies of the P­(DTP-Ph-Pyr)/Calixarene-AuNP (0.75 mg of calixarene)/Cyanobacteria (500 mg/mL) photoanode structure with different cycles for the conducting polymer film: (a) 20, (b) 40, (c) 60, (d) 80, (e) 100. All chronoamperometry studies were done in phosphate buffer (pH = 7.4) and under visible light at 1400 W/m^2^ power.

After the control experiments for photocurrent generation, studies were performed to find the optimum conditions for producing a high photocurrent. First, the amount of calixarene for the Calixarene-AuNP suspension was optimized. The P­(DTP-Ph-Pyr) film was coated on the gold electrode by applying 40 cycles. The Calixarene-AuNP suspensions containing 0.25, 0.5, 0.75, and 1.0 mg of calixarene were dropped onto the polymer film. Then, 750 mg/mL cyanobacteria were immobilized onto the P­(DTP-Ph-Pyr)/Calixarene-AuNP-modified gold electrode. The photoanode electrode configuration was immersed in the buffer solution and exposed to visible light, resulting in the generation of photocurrents with varying magnitudes. An increase in the calixarene concentration resulted in a corresponding enhancement of the photocurrent generated by the system. This trend persisted up to a concentration of 0.5 mg. However, beyond 0.75 mg, no further increase in the photocurrent was observed, suggesting that the maximum electron transfer efficiency had been reached under these conditions. In spite of the rise in the calixarene amount for the 1.0 mg, the speed of electron transfer decreased, and the photocurrent generated started to decrease. This phenomenon can be attributed to the sufficient covalent interactions established between the Calixarene-AuNP complex and the cyanobacterial cells along with the progressively increasing complexity of the electron conduction pathways as the calixarene concentration increased within the system. Conversely, an insufficient amount of calixarene may fail to establish an optimal microenvironment conducive to efficient electron transfer. Within the scope of this optimization study, 0.75 mg was identified as the optimal calixarene concentration for achieving maximum performance. (Figure 1D).

An additional characterization study for the photoanode electrode focused on determining the optimal concentration of the cyanobacteria. In this study, the Calixarene-AuNP suspension containing 0.75 mg of calixarene was coated onto the P­(DTP-Ph-Pyr) polymer film, which was prepared by using 40 electropolymerization cycles. Subsequently, cyanobacteria at concentrations of 250, 500, 750, and 1000 mg/mL were immobilized on the P­(DTP-Ph-Pyr)/Calixarene-AuNP-modified gold electrode. The results showed that the photocurrent generated reached its maximum at a cyanobacteria concentration of 500 mg/mL. Beyond this concentration, the photocurrent decreased due to the thickening of the biocomponent layer, which hindered electron transfer to the electrode. This reduction is attributed to water oxidation during photosynthesis (Figure 1E). Therefore, 500 mg/mL was determined to be the optimal concentration of cyanobacteria for use in the photoanode.

Another optimization effort for the photoanode focused on determining the optimal thickness of the polymer film. While all experiments in the preceding study employed a polymer film synthesized through 40 electropolymerization cycles, the current study involved the electrochemical polymerization of the (DTP-Ph-Pyr) monomer onto bare gold electrodes using 20, 40, 60, 80, and 100 cycles to assess the effect of film thickness on device performance. A Calixarene-AuNP suspension comprising 0.75 mg of calixarene was subsequently applied as a top coating onto the polymer films fabricated through the respective electropolymerization cycle numbers. Subsequently, the immobilization of cyanobacteria at a concentration of 500 mg/mL was carried out on the modified electrode surface. Upon immersion of the photoanode in phosphate buffer (pH 7.4) and exposure to visible light, the highest photocurrent response was recorded for polymer films synthesized through 60 electropolymerization cycles. A decline in photocurrent was observed when polymer electrodes fabricated with 80 and 100 electropolymerization cycles were employed. Beyond 60 cycles, the increased thickness of the polymer film impeded efficient electron transfer, thereby limiting the number of electrons reaching the electrode surface (Figure 1F). Accordingly, this number of electropolymerization cycles was identified as the optimal condition for polymer film formation.

After the optimization studies, it was understood that the impact of the Calixarene-AuNP structure on device performance is significantly greater than that of the conductive DTP-Ph-Pyr polymer. This can be explained by the unique features of the calixarene and the AuNPs. Owing to their distinct amphiphilic nature—possessing both hydrophilic and hydrophobic domains, calixarenes are capable of engaging in host–guest interactions with a diverse spectrum of molecular species. These features facilitate efficient cyanobacteria loading and provide an ideal site for selective binding with guest molecules, such as cyanobacteria, through covalent bonding. Besides, gold nanoparticles (AuNPs) were employed for the specific binding of calixarenes as a consequence of their large active surface area, excellent biocompatibility, and high conductivity, which supports rapid electron transfer. However, the high effect of conductive P­(DTP-Ph-Pyr) on the device performance should not also be overlooked, as it significantly contributes to rapid electron transfer and serves a pivotal function in the high photocurrent generation of the constructed BPV.

### 3.3 BPV Characterization and Photosynthetic Hydrogen Generation Studies

After the conditions for the anode of the BPV were optimized, it was subjected to repeated illumination cycles by applying a constant potential of 0 V under steady illumination at 1400 W/m^2^. A progressive decline in photocurrent was detected over consecutive measurement cycles. This decline was attributed to the accumulation of oxygen gas, a byproduct of water oxidation during photosynthesis, which impeded the forward electron transfer rate responsible for generating the photocurrent. The bilirubin oxidase (BOx) enzyme was introduced into the electrolyte (buffer solution) at a concentration of 1.5 mg/mL to prevent this negative interference. The role of the BOx enzyme was to convert the oxygen gas produced during photosynthesis back into water, effectively preventing the photocurrent reduction caused by oxygen accumulation.^31,44^ As a result, the BPV maintained a stable photocurrent during on–off illumination cycles, underscoring the critical role of managing water oxidation byproducts in ensuring BPV efficiency and stability (Figure 2A). The operational stability of the BPV was determined via continuous chronoamperometric photocurrent measurements conducted for 600 min. The BPV maintained 95% of its initial photocurrent activity after 240 min, 80% after 300 min, 54% after 360 min, 21% after 420 min. The results indicated that the BPV sustained high photocurrent activity for a long duration of time without any notable decrease thereafter (Figure 2B). This signifies that the BPV demonstrates exceptional operational stability and elevated intraelectrode repeatability.

**Figure 2:**
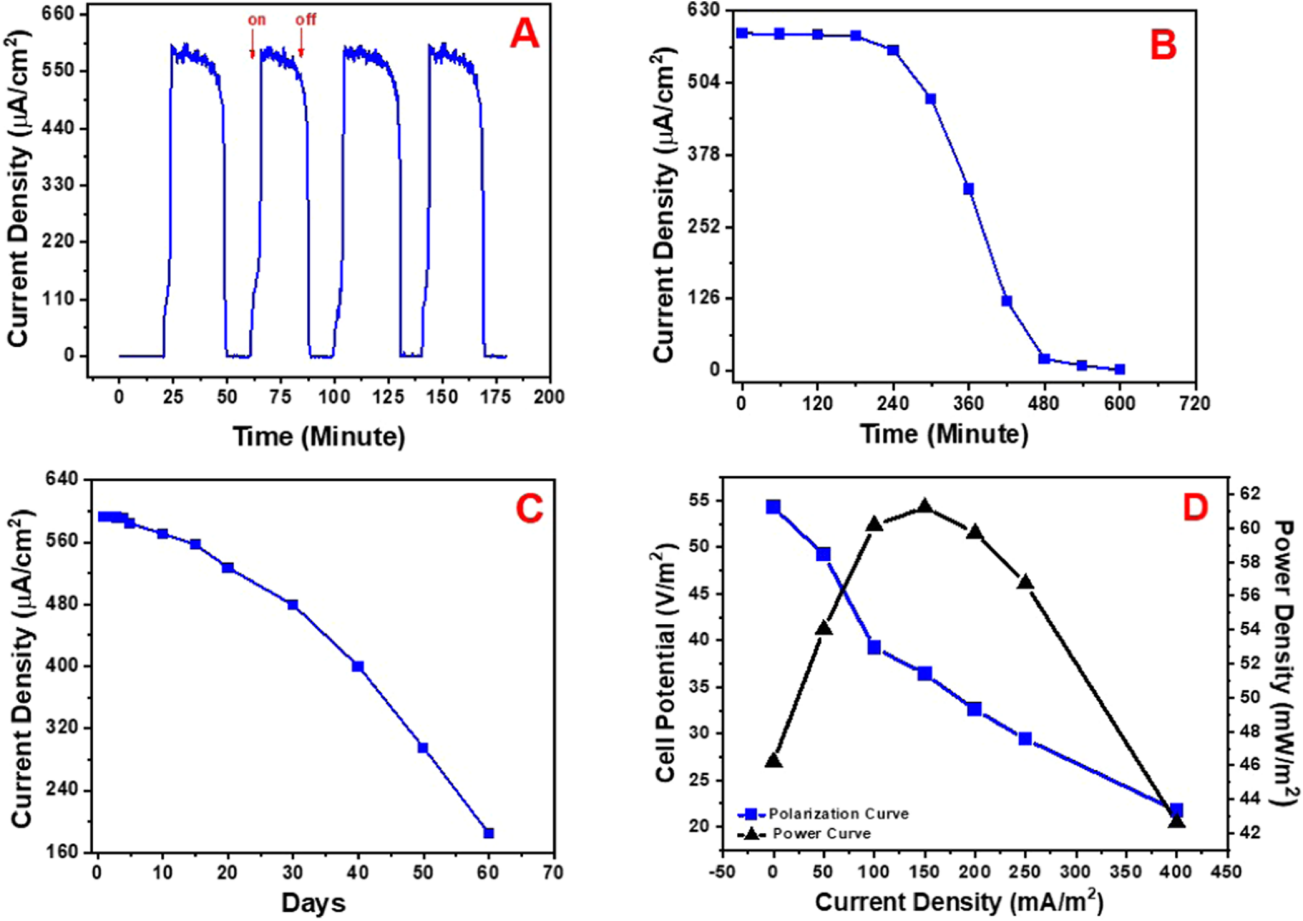
(A) Intraelectrode repeatability; (B) working stability; (C) storage stability; (D) polarization and power output curves of the BPV solar cell. (Au Electrode/P­(DTP-Ph-Pyr)/Calixarene-AuNP/Cyanobacteria type anode electrode and Au Electrode/P­(DTP-Ph-NH_2_)/PtNP-type cathode electrodes were used during all of these studies).

The storage stability of P­(DTP-Ph-Pyr)/Calixarene-AuNP/Cyanobacteria-based BPV was investigated over a period of 60 days, with photocurrent activity recorded daily for the initial 5 days and subsequently every 5 days. During the initial 15 days, there was no noticeable decrease in the level of photocurrent generation. The BPV maintained 90% of its initial photocurrent activity after 20 days, 81% after 30 days, 69% after 40 days, 50% after 50 days, and 31% by the conclusion of 60 days. This verifies that the BPV has robust storage stability (Figure 2C). The reason for the decrease in the photocurrent generation performance of BPV as the number of days increases in the storage stability experiments of BPV, can be explained that the cyanobacteria, which are living whole cells, get older day by day and a significant part of their cells begin to die; therefore, their photosynthetic activity begins to decrease. The less photosynthesis done by cyanobacteria, the less photocurrent generated by the BPV. The BPV system’s electricity generation was also evaluated using the polarization curve method, which involved using resistors connected in series with resistance values varying between 100 Ω and 10 kΩ under visible light illumination. The BPV reached its maximum power generation of 62 mW/m^2^ at a 150 mA/m^2^ current density under pseudosteady-state conditions (Figure 2D).

The hydrogen generation process within the BPV system was carried out under varying illumination durations (2.5, 5, 10, 15, 20, 25, and 30 min) at a light intensity of 1400 W/m^2^ (Scheme 3). For hydrogen gas analysis, a 1 mL (cm^3^) gas sample was extracted from the system by using an injector and analyzed via gas chromatography. The quantification revealed that, under 1400 W/m^2^ illumination for 30 min, the BPV solar cell produced 34.2 × 10^–8^ ± 1.05 mol/cm^3^ (342 μmol/L) (with 3.07% error) of hydrogen gas, achieving a quantum efficiency of 9.6% (Figure 3A). This procedure was repeated three times, yielding consistent results, and statistical data for hydrogen generation after 30 min illumination are given in the Supporting Information as Table S1.

**Figure 3:**
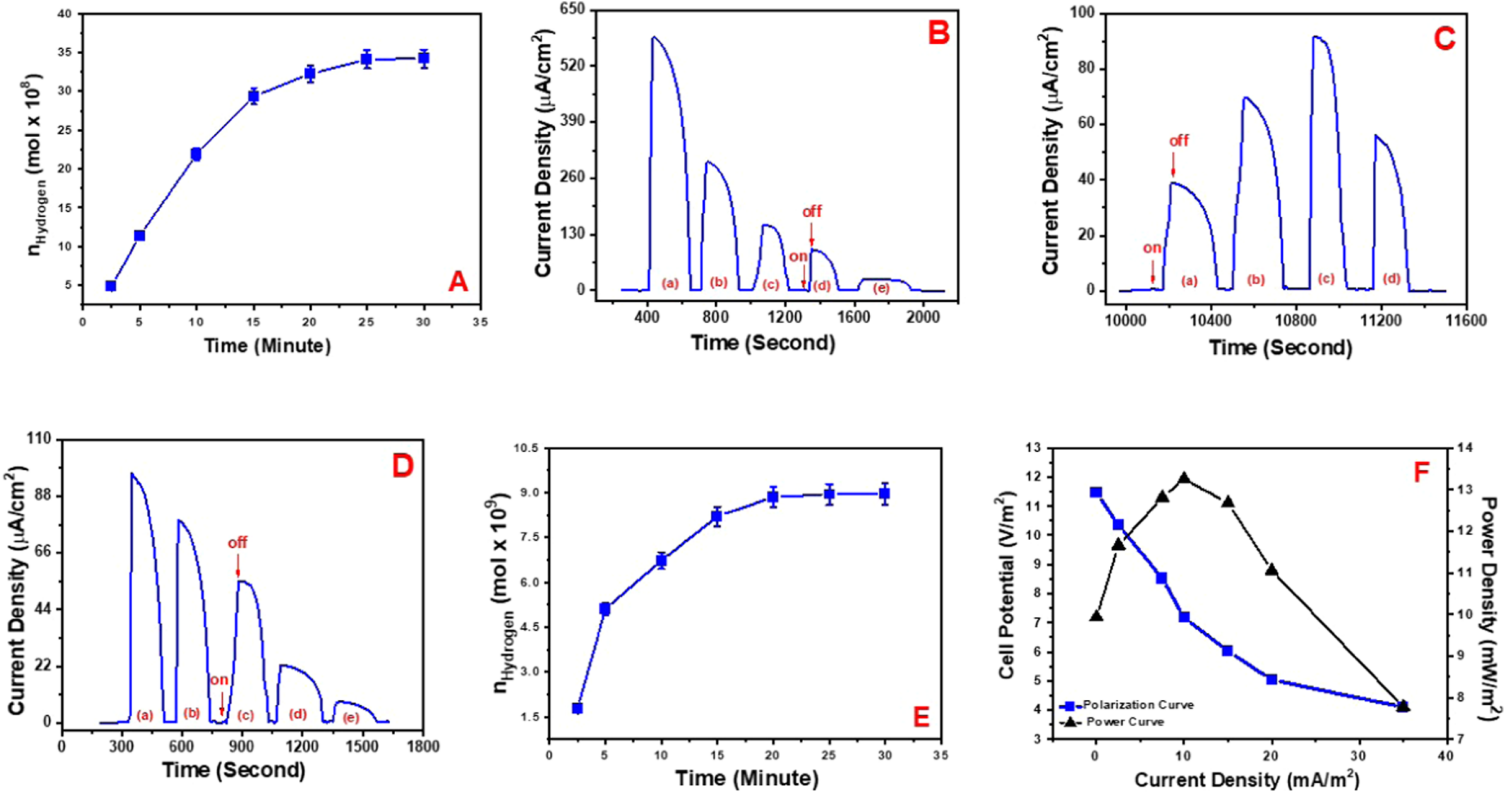
(A) Temporal hydrogen gas generation via the BPV solar cell. (B) Chronoamperometric on–off analysis of photocurrent responses generated by the BPV solar cell via water oxidation, driven by photosynthetic activity, was conducted in the presence of increasing concentrations of 3-(3,4-dichlorophenyl)-1,1-dimethylurea (DCMU), specifically (a) 0, (b) 0.2, (c) 0.4, (d) 0.8, and (e) 1.5 mM. All measurements were performed in aqueous phosphate buffer solution (pH 7.4) under visible light illumination with an intensity of 1400 W/m^2^. (C) Photocurrent outputs generated by the BPV solar cell were evaluated in the presence of varying glucose concentrations: (a) 2 mM, (b) 4 mM, (c) 6 mM, and (d) 8 mM. All experiments were conducted under a constant applied potential of 0 V and visible light illumination at an intensity of 1400 W/m^2^, in the presence of 1.6 mM 3-(3,4-dichlorophenyl)-1,1-dimethylurea (DCMU). (D) Chronoamperometric on–off analysis of the BPV solar cell was performed in the presence of 1.6 mM DCMU and 6 mM glucose, with incremental concentrations of iodoacetate: (a) 0, (b) 0.2, (c) 0.4, (d) 0.8, and (e) 1.0 mM. All experiments were conducted in phosphate buffer solution (pH 7.4) under visible light illumination at an intensity of 1400 W/m^2^. (E) Time-dependent hydrogen gas generated by the BPV solar cell via respiratory system of the cyanobacteria. (F) Polarization and power curves of the BPV solar cell via the respiratory system of the cyanobacteria. (Au Electrode/P­(DTP-Ph-Pyr)/Calixarene-AuNP/Cyanobacteria type anode electrode and Au Electrode/P­(DTP-Ph-NH_2_)/PtNP-type cathode electrodes were used during all of these studies).

Table 1 provides a comparative analysis of the BPV cell’s performance in hydrogen production via water splitting under visible light with findings from relevant literature.^31,34,45–47^ The cyanobacteria-based BPV system demonstrated remarkable hydrogen production performance, generating 342 μmol/L hydrogen gas, comparable to results reported in previous studies. These findings highlight the BPV cell’s high efficiency in hydrogen production.

**Table 1:** Comparison of Photoelectrochemical Water Splitting Performance Based on H_2_ Gas Generation

material	light source	H_2_ gas concentration (μmol/L)	references
P(DTP-Ph-Pyr)/calixarene-AuNP/cyanobacteria	visible light region	342	this work
calixarene/cyanine dye/IrO2NP	visible light region	125	^31^
Gr-cellulose/cyanobacteria	visible light region	295	^34^
LaFeO_3_/Fe_2_O_3_	visible light region	80	^45^
SnO_2_/TiO_2_	visible light region	45	^46^
conducting polymer/AuNP/cyanobacteria	visible light region	265	^47^

### 3.4 Respiratory Photocurrent and Hydrogen Generation Studies

Through the mechanism of oxygenic photosynthesis, these organisms capture sunlight to oxidize water and reduce carbon dioxide, ultimately generating molecular oxygen and glucose as the primary products. These organisms also possess a respiratory apparatus that operates concurrently with the photosynthetic machinery, enabling the metabolism of sugars. Within chlorophyll, Photosystem I (PS I) plays a pivotal role in this process by contributing to the oxidation of carbon compounds in the respiratory systems of cyanobacteria, even in the absence of photosynthetic activity. The inhibition of Photosystem II (PS II) in cyanobacteria can be effectively achieved by introducing 3-(3,4-dichlorophenyl)-1,1-dimethylurea (DCMU) into the environment surrounding the anode. DCMU suppresses PS II activity, thereby inhibiting photosynthesis. The addition of DCMU to the biophotovoltaic (BPV) cell led to a measurable decline in photocurrent generation. This reduction occurred because DCMU blocks the activity of PS II, which is essential for photosynthetic electron transfer. As the concentration of DCMU increased, the photocurrent generated by the BPV cell progressively diminished and eventually ceased after a certain threshold (Figure 3B). However, in the presence of 1.6 mM DCMU, the addition of glucose to the BPV system, with concentrations increasing from 2 to 8 mM (Figure 3B), resulted in a significant enhancement of the photocurrent. This increase is attributed to improved electron transfer, driven by the cyanobacteria’s ability to oxidize and metabolize glucose through their respiratory processes.

In the presence of 6 mM glucose, the photocurrent generated by the biophotovoltaic (BPV) system increased significantly, reaching a peak value of 91 μA. However, a further increase in glucose concentration to 8 mM resulted in a decline in the photocurrent, identifying 6 mM as the optimal glucose concentration for maximum photocurrent generation (Figure 3C). To verify that the observed photocurrent in the presence of DCMU and glucose was attributed to the respiratory activity of cyanobacteria, iodoacetate, an inhibitor of the respiratory system, was employed. When iodoacetate was introduced at concentrations of 0.2, 0.4, 0.8, and 1.0 mM into the BPV cell under 1400 W/m^2^ light illumination with 1.6 mM DCMU and 6 mM glucose, the photocurrent generated via the respiratory system of the cyanobacteria was markedly reduced (Figure 3D). These results unequivocally demonstrate that the cyanobacterial respiratory system is the primary electron donor for photocurrent generation under these experimental conditions. The power output of the BPV system driven by the respiratory pathway was assessed using polarization curves. Resistors with resistance values spanning from 100 Ω to 10 kΩ were connected in series, and measurements were conducted under 1400 W/m^2^ visible light illumination with 1.6 mM DCMU and 6 mM glucose. The system’s capacity for hydrogen gas production through the respiratory activity of cyanobacteria was evaluated under varying durations of illumination (2.5, 5, 10, 15, 20, 25, and 30 min) using a solar simulator emitting light at an intensity of 1400 W/m^2^. In the presence of 1.6 mM DCMU and 6 mM glucose, hydrogen gas production reached 8.95 × 10^–9^ ± 0.45 mol/cm^3^ (with 5.0% error) (8.95 μmol/L) after 30 min of illumination (Figure 3E). The study was repeated three times, and the results were found to be reproducible and statistical data for hydrogen generation after 30 min illumination is given in the Supporting Information as Table S1. Furthermore, the maximum power output of the BPV system under pseudosteady-state conditions was recorded as 13.2 mW/m^2^, corresponding to a current density of 10 mA/m^2^ (Figure 3F). The quantum efficiency for hydrogen generation was also calculated to be 4.8%. Photosynthesis uses light energy to drive electron excitation, allowing for direct utilization of high-energy photons. However, respiration uses chemical energy from organic molecules, meaning energy has already been partially lost in previous metabolic steps, reducing the overall efficiency of electron transfer per input energy The lower quantum efficiency of respiration compared to quantum efficiency of photosynthesis is mainly due to less efficient electron transfer mechanisms, lower energy inputs per electron, and greater energy dissipation as heat. Photosynthesis is optimized for light energy conversion, making it inherently more efficient than respiration in terms of quantum yield per absorbed photon. To increase the quantum efficiency of respiration in the BPV system, a multifaceted approach can be taken. This includes enhancing electron transfer pathways, optimizing metabolic processes, improving the anode-cyanobacteria interface, reducing internal energy losses, and using synthetic biology tools to engineer high-efficiency electron transport systems. By implementing these strategies, in the future of the BPV works, higher photocurrent and hydrogen production can be achieved, making the BPV system more efficient and sustainable.

## 4 Conclusions

This study introduces a novel approach for advancing the field of biological photovoltaic solar cells (BPVs) by leveraging the respiratory functions of cyanobacteria to enable the concurrent production of photocurrent and hydrogen. Unlike traditional BPVs that primarily depend on photosynthesis, this research investigates the underutilized potential of cyanobacterial respiration, thereby presenting innovative pathways for sustainable energy generation. Central to this innovation is the development of a novel photoanode design comprising a P­(DTP-Ph-Pyr)/Calixarene-AuNP/Cyanobacteria. Initially, a novel pyrrole-functionalized DTP-type conducting polymer was developed, followed by using an original synthesized calixarene and AuNPs to enhance the photoanode performance of the BPV. P­(DTP-Ph-Pyr) was employed to enhance the overall performance of the photoanode and to facilitate more efficient charge transfer characteristics. This conductive polymer has a pyrrole moiety that can do stronger π–π stacking interactions, improved charge carrier mobility, and superior light absorption, facilitating more efficient electron transport. Hence, the hydrogen and photocurrent generation capabilities of the BPV were improved. Because of its amphiphilic architecture—characterized by a hydrophilic outer surface and a hydrophobic inner cavity, the calixarene utilized in this study facilitated improved interaction with cyanobacteria. This structural feature not only enhanced bacterial immobilization efficiency but also provided a favorable platform for selective covalent binding with cyanobacterial cells as guest entities. In addition to their outstanding physicochemical properties, gold nanoparticles (AuNPs) were employed to facilitate the specific attachment of cyanobacteria onto the surface, owing to their high specific surface area, superior biocompatibility, and excellent electrical conductivity, all of which contribute to increase the speed of electron transfer. Thiol functional groups acted as anchoring sites, enabling the covalent immobilization of calixarene onto AuNPs through the formation of stable Au–S bonds. Taking into account the synergistic contributions of conducting polymers, calixarene, and AuNPs, it was observed that the role of these combinations was very effective and positively correlated to the performance of the photoanode. Furthermore, a significant innovation of this study is the design of our BPV system, which not only generates energy but also provides a solution for hydrogen production. This study illustrates the viability of employing cyanobacterial respiration for sustainable energy generation and underscores materials science as a crucial foundation for enhancing BPV technology. This study also highlights the potential of cyanobacteria to produce electricity and hydrogen by taking advantage of their respiratory properties under conditions where photosynthesis is limited. Our methodical design strategy, combined with streamlined and advanced production techniques, demonstrates potential in advancing BPVs toward a more environmentally friendly and sustainable future.

## Supplementary Material



## Notes

Detailed information about incubation and characterization of *Leptolyngbia* sp. cyanobacteria; synthesis, characterization, and homopolymerization of DTP-Ph-Pyr monomer; and synthesis and characterization of PtNPs (PDF)

## References

[1] Lewis N. S., Nocera D. G. (2006). Powering the planet: Chemical challenges in solar energy
utilization. Proc. Natl. Acad. Sci. U.S.A..

[2] Logan B. E., Hamelers B., Rozendal R., Schroder U., Keller J., Freguia S., Aelterman P., Verstraete W., Rabaey K. (2006). Microbial Fuel Cells: Methodology
and Technology. Environ. Sci. Technol..

[3] McCormick A. J., Bombelli P., Scott A. M., Philips A. J., Smith A. G., Fisher A. C., Howe C. J. (2011). Photosynthetic
biofilms in pure culture
harness solar energy in a mediatorless bio-photovoltaic cell (BPV)
system. Energy Environ. Sci..

[4] Buyukharman M., Gover T., Gumus A., Gumus S., Yildiz H. B. (2024). Design
of a Novel Green Algae-Based Biological Photovoltaic Cell with High
Photocurrent and a Photoelectrochemical Biosensing Approach Utilizing
the BPV for Pesticide Analysis in Water. ChemistrySelect.

[5] Rosenbaum M., Schroder U. (2010). Photomicrobial Solar and Fuel Cells. Electroanalysis.

[6] Pankratova G., Bollella P., Pankratov D., Gorton L. (2022). Supercapacitive biofuel
cells. Curr. Opin. Biotechnol..

[7] Buyukharman M., Mulazımoglu I. E., Yildiz H. B. (2024). Construction of a Conductive Polymer/AuNP/Cyanobacteria-Based
Biophotovoltaic Cell Harnessing Solar Energy to Generate Electricity
via Photosynthesis and Its Usage as a Photoelectrochemical Pesticide
Biosensor: Atrazine as a Case Study. ACS Omega.

[8] Tanvir R. U., Zhang J., Canter T., Chen D., Lu J., Hu Z. (2021). Harnessing solar energy
using phototrophic microorganisms: A sustainable
pathway to bioenergy, biomaterials, and environmental solutions. Renewable Sustainable Energy Rev..

[9] Nishio K., Hashimoto K., Watanabe K. (2013). Light/electricity conversion by defined
cocultures of Chlamydomonas and Geobacter. J.
Biosci. Bioeng..

[10] Tucci M., Grattieri M., Schievano A., Cristiani P., Minteer S. D. (2019). Microbial amperometric biosensor
for online herbicide
detection: Photocurrent inhibition of Anabaena variabilis. Electrochim. Acta.

[11] Bombelli P., Bradley R. W., Scott A. M., Philips A. J., McCormick A. J., Cruz S. M., Anderson A., Yunus K., Bendall D. S., Cameron P. J., Davies J. M., Smith A. G., Howe C. J., Fisher A. C. (2011). Quantitative analysis
of the factors limiting solar
power transduction by *Synechocystis* sp. PCC 6803
in biological photovoltaic devices. Energy Environ.
Sci..

[12] Lund S., Wey L. T., Peltonen J., Bobacka J., Latonen R. M., Allahverdiyeva Y. (2024). Graphene and
graphene–cellulose nanocrystal
composite films for sustainable anodes in biophotovoltaic devices. Sustainable Energy Fuels.

[13] Cevik E., Buyukharman M., Yildiz H. B. (2019). Construction of efficient bioelectrochemical
devices: Improved electricity production from cyanobacteria (*Leptolyngbia* sp.) based on π-conjugated conducting
polymer/gold nanoparticle composite interfaces. Biotechnol. Bioeng..

[14] Hasan K., Yildiz H. B., Sperling E., Conghaile P.´O., Packer M. A., Leech D., Hagerhall C., Gorton L. (2014). Photo-electrochemical communication between cyanobacteria
(*Leptolyngbia* sp.) and osmium redox polymer modified
electrodes. Phys. Chem. Chem. Phys..

[15] Gutsche C. D., Muthukrishnan R. (1978). Analysis of the Product Mixtures Produced by the Base-Catalyzed
Condensation of Formaldehyde with Para-Substituted Phenols. J. Org. Chem..

[16] Ren H., Wang H., Wen W., Li S., Li N., Huo F., Yin C. (2023). A summary of calixarene-based fluorescent sensors developed
during the past five years. Chem. Commun..

[17] Sayin S., Azak H., Yildiz H. B., Camurlu P., Akkus G. Uysal., Toppare L., Ersoz M. (2015). Calixarene
assembly with enhanced
photocurrents using P­(SNS-NH2)/CdS nanoparticle structure modified
Au electrode systems. Phys. Chem. Chem. Phys..

[18] Li S., Xiao Z., Li J.-J., Hu Z.-Y., Yang Y., Kan B., Guo D.-S., Wan X., Yao Z., Li C., Chen Y. (2023). Calixarenes enabling well-adjusted organic-inorganic interface for
inverted organic solar cells with 18.25% efficiency and multifold
improved photostability under max power point tracking. Science China Chemistry.

[19] Chang X., Xu Y., von Delius M. (2024). Recent advances
in supramolecular fullerene chemistry. Chem.
Soc. Rev..

[20] Nug R., Rao C. P. (2022). Calixarene-mediated
host–guest interactions
leading to supramolecular assemblies: visualization by microscopy. Chem. Commun..

[21] Geng Y., Tang A., Tajima K., Zeng Q., Zhou E. (2019). Conjugated
materials containing dithieno­[3,2-b:2′,3′-d]­pyrrole
and its derivatives for organic and hybrid solar cell applications. J. Mater. Chem. A.

[22] Cao J., Du F., Yang L., Tang W. (2020). The design of dithieno­[3,2-b:2′,3′-d]­pyrrole
organic photovoltaic materials for high-efficiency organic/perovskite
solar cells. J. Mater. Chem. A.

[23] Nguyen T. H., Nguyen T. A., Tran H. M., Nguyen L-T.T., Luu A. T., Lee J. Y., Nguyen H. T. (2017). N-Benzoyl
dithieno­[3,2-b:2′,3′-d]­pyrrole-based
hyperbranched polymers by direct arylation polymerization. Chem. Cent. J..

[24] Bezgin
Carbas B., Yildiz H. B. (2024). A review of dithieno­[3,2-b:2′,3′-d]­pyrrole-based
electrochromic conjugated polymers. Eur. Polym.
J..

[25] Fortsch S., Mena-Osteritz E., Bauerle P. (2021). Synthesis and characterization of
β,β′-dimethylated dithieno­[3,2-b:2′,3′-d]­pyrroles
and their corresponding regioregular conducting electropolymers. Polym. Chem..

[26] Chen X., Shen S., Guo L., Mao S. S. (2010). Semiconductor-based
Photocatalytic Hydrogen Generation. Chem. Rev..

[27] Chen Y., Wang Y., Xu H., Xiong G. (2008). Efficient production
of hydrogen from natural gas steam reforming in palladium membrane
reactor. Appl. Catal., B.

[28] Gaudillere C., Navarrete L., Serra J. M. (2014). Syngas production at intermediate
temperature through H2O and CO2 electrolysis with a Cu-based solid
oxide electrolyzer cell. Int. J. Hydrogen Energy.

[29] Hydrogen Production from Nuclear Energy; Naterer, G. F. ; Dincer, I. ; Zamfirescu, C. , Eds.; Springer London: London, 2013.

[30] Turn S., Kinoshita C., Zhang Z., Ishimura D., Zhou J. (1998). An experimental
investigation of hydrogen production from biomass gasification. Int. J. Hydrogen Energy.

[31] Tekin M., Cevik E., Sayin S., Yildiz H. B. (2020). Photocurrent and
hydrogen production by overall water splitting based on polymeric
composite Calix­[n]­arene/Cyanin Dye/IrO2 nanoparticle. Int. J. Hydrogen Energy.

[32] McCormick A. J., Bombelli P., Lea-Smith D. J., Bradley R. W., Scott A. M., Fisher A. C., Smith A. G., Howe C. J. (2013). Hydrogen production
through oxygenic photosynthesis using the cyanobacterium *Synechocystis* sp. PCC 6803 in a bio-photoelectrolysis cell (BPE) system. Energy Environ. Sci..

[33] Saper G., Kallmann D., Conzuelo F., Zhao F., T́oth T. N., Liveanu V., Meir S., Szymanski J., Aharoni A., Schuhmann W., Rothschild A., Schuster G., Adir N. (2018). Live cyanobacteria produce photocurrent
and hydrogen using both the respiratory and photosynthetic systems. Nat. Commun..

[34] Atakhanov A., Ashurov N., Erkartal M., Yildiz H. B. (2024). Photoelectrochemical
Communication Between Cyanobacteria and Electrospun Cellulose Acetate–Graphene-Based
Electrodes for Photosynthetic and Respiratorial Photocurrent and Hy-drogen
Generations via Sustainable Solar Energy. Solar
RRL.

[35] Ha J. M., Solovyov A., Katz A. (2009). Langmuir, Synthesis and Characterization
of Accessible Metal Surfaces in Calixarene-Bound Gold Nanoparticles. Langmuir.

[36] Demirkol D. Odaci., Yildiz H. B., Sayin S., Yilmaz M. (2014). Enzyme immobilization
in biosensor constructions: self-assembled monolayers of calixarenes
containing thiols. RSC Adv..

[37] Yildiz H. B., Tel-Vered R., Willner I. (2008). Solar Cells with Enhanced Photocurrent
Efficiencies Using Oligoaniline-Crosslinked Au/CdS Nanoparticles Arrays
on Electrodes. Adv. Funct. Mater..

[38] Bezgin
Carbas B., Ergin N. M., Yildiz H. B., Kivrak A. (2024). Electrochemical
and optical properties of poly­(4-(4-(1H-pyrrol-1-yl)­phenyl)-4Hdithieno­[3,2-b:2’,3′-d]­pyrrole. Polym. Bull..

[39] Li Z.-T., Ji G. Z., Zhao C. X., Yuan S. D., Ding H., Huang C., Du A. L., Wei M. (1999). Self-Assembling
Calix­[4]­arene
[2]­Catenanes. Preorganization, Conformation, Selectivity, and Efficiency. J. Org. Chem..

[40] Sayin S., Yildiz H. B., Eymur S. (2018). Synthesis of Various Calix[4]­arene
Derivatives with Mercaptoalkyl Chains and Their Application in Removing
Cr­(VI) from Aqueous Solution. Polycyclic Aromatic
Compunds.

[41] Soylemez S., Kanik F. E., Nurioglu A. G., Akpinar H., Toppare L. (2013). A novel conducting
copolymer: Investigation of its matrix properties for cholesterol
biosensor applications. Sensors and. Actuators,
B:Chemical.

[42] Udum Y. A., Yildiz H. B., Azak H., Sahin E., Talaz O., Çırpan A., Toppare L. (2014). Synthesis and spectroelectrochemistry
of dithieno­(3,2-b:2′,3′ -d)­pyrrole derivatives. J. Appl. Polym. Sci..

[43] Tel-Vered R., Yildiz H. B., Yan Y. M., Willner I. (2010). Plugging into Enzymes
with Light: Photonic ‘Wiring’ of Enzymes with Electrodes
for Photobiofuel Cells. Small.

[44] Yan Y. M., Baravik I., Tel-Vered R., Willner I. (2009). An Ethanol/O2 Biofuel
Cell Based on an Electropolymerized Bilirubin Oxidase/Pt Nanoparticle
Bioelectrocatalytic O2-Reduction Cathode. Adv.
Mater..

[45] Yu Q., Meng X. G., Wang T., Li P., Liu L. Q., Chang K., Liu G. G., Ye J. H. (2015). A highly durable
p-LaFeO3/n-Fe2O3 photocell for effective water splitting under visible
light. Chem. Commun..

[46] Alibabaei L., Sherman B. D., Norris M. R., Brennaman M. K., Meyer T. J. (2015). Visible photoelectrochemical water
splitting into H_2_ and O_2_ in a dye-sensitized
photoelectrosynthesis
cell. Proc. Natl. Acad. Sci. (PNAS).

[47] Carbas B. Bezgin., Guler M., Yucel K., Yildiz H. B. (2023). Construction of
novel cyanobacteria-based biological photovoltaic solar cells: Hydrogen
and photocurrent generated via both photosynthesis and respiratory
system. J. Photochem. Photobiol., A.

