# Supplementary material for: A Novel Architecture Based on a Pyrrole-Functionalized Dithieno[3,2-b:2′,3′-d]pyrrole (DTP)-Type Conducting Polymer and Thiol-Modified Calixarene Derivative for Biophotovoltaic Solar Cells: Photocurrent and Hydrogen Generations via Both Photosynthesis and Respiratory System
Source: ACS Omega. 2025 May 23;10(21):21450–62. doi: 10.1021/acsomega.5c00164 (PMC12138618; doi:10.1021/acsomega.5c00164)
Supplement: Supplementary file 1 [file ao5c00164_si_001.pdf]

A Novel Architecture Based On a Pyrrole Functionalized Dithieno[3,2-b:2′,3′-d]pyrrole (DTP) Type Conducting Polymer and Thiol Modified Calixarene Derivative for Biophotovoltaic Solar Cells: Photocurrent and Hydrogen Generations via Both Photosynthesis and Respiratory System

Mustafa Buyukharman[1], Huseyin Bekir Yildiz[2], Sumeyye Bakim[3], Mustafa Esen Marti[4,*]

[1]Department of Physics, Institute of Graduate Studies in Science, Istanbul University, 34134 Istanbul, Turkey

[2]Department of Electrical-Electronics Engineering, Faculty of Engineering and Natural Sciences, KTO Karatay University, 42020 Konya, Turkey

[3]Department of Computer Engineering, Faculty of Engineering and Natural Sciences, KTO Karatay University, 42020 Konya, Turkey

[4]Department of Chemical Engineering, Faculty of Engineering and Natural Sciences, Konya Technical University, 42250 Konya, Turkey

*Corresponding Author: Mustafa Esen Marti (email: memarti@ktun.edu.tr)

Incubation and Characterization of Leptolyngbia Sp. Type Cyanobacteria

Leptolyngbia sp. type of cyanobacteria, among photosynthetic microorganisms, was purchased from Carolina™ (USA). The cyanobacteria used in this study were reproduced according to the literature [1]. Modified Leonian agar (MLA) complex, used in previous studies and generally preferred for green algae, was used as the medium. Incubation was carried out at room temperature in a low ion intensity environment and a white fluorescence lamp with a photon power of 40 µmol, was adjusted to 12:12 light/dark. Cells were centrifuged at 20 ℃ for 10 min at 4000 rpm, washed with electrolyte and then centrifuged under the same conditions. The obtained Leptolyngbia sp. cells were resuspended with the same electrolyte solution (1 g/ml) and immediately used for photoelectrochemical measurements. The total amount of chlorophylls (chlorophyll a and chlorophyll b) on cyanobacteria will be determined by using the method of Porra et al. [2]. According to this method, 10 µL cyanobacteria suspensions will be mixed with 990 µL % 80 acetone and then centrifuged at 14.000 rpm for 10 sec., and the absorbance of the supernatant will be determined at 645 nm and 663 nm. The amounts of chlorophylls will be calculated in accordance with the formula below.

Chlorophyll a (mg/mL) = 12.7A663 - 2.69A645

Chlorophyll b (mg/mL) = 22.9A645 - 4.68A663

Total Chlorophyll (mg/mL) = Chlorophyll a + Chlorophyll b

Homopolymerization of DTP-Ph-Pyr monomer

The electrochemical behavior of DTP-Ph-Pyr monomer was investigated by cyclic voltammetry (CV) in an electrolyte solution of 0.1 M $TBAPF_6$ dissolved in DCM. (Figure SI-1 a). As seen from the first cycle of resulting voltammogram, DTP-Ph-Pyr monomer had an irreversible broad anodic oxidation peak at 0.6 V and a sharp peak 1.30 V versus Ag/AgCl. The oxidation potential value of monomer is similar to its analogs [2]. The electrochemical polymerization of monomer was achieved in an electrolyte solution consisting of 0.1 M TBAPF6, respectively, dissolved in DCM via repetitive cycling. (Figure S1-a). It was observed that the anodic and cathodic current values increased with each increasing peak in CV data, indicating that a polymeric film was formed on the working electrode surface. For electrochemical behavior analysis of polymeric film, DTP-Ph-Pyr monomer coated on Pt disc electrode (25 cycled) was cleaned with DCM solvent to remove monomeric and oligomeric species. Afterwards, P(DTP-Ph-Pyr) film coated on Pt disc was inserted in the CV cuvette with other electrodes in 0.1 M TBAPF6/ACN medium (Figure S1-b). Inspection of Figure S1-b

shows that a clear reversible redox couple at 0.97 V (doped) and 0.37 V (de-doped) were observed with a scan rate of 100 mV/s [3]. If you notice that Electrochemical polymerization of DTP-Ph-Pyr monomer occurred in the solution medium of Tetrabutylammonium hexafluorophosphate /Dichloromethane (TBAPF6/DCM) (Figure S1a and Electrochemical characterization of P(DTP-Ph-Pyr) polymer film was done in the medium of Tetrabutylammonium hexafluorophosphate/Acetonitrile (TBAFP6/ACN) (FigureS1b). If the ionic conductivity of the electrolyte is high, the charge transfer between the electrode surface and the solution becomes faster, which can increase the charge density. Acetonitrile is more conductive than dichloromethane and therefore the current density shown in Figure S1a is higher than that of Figure S1b.

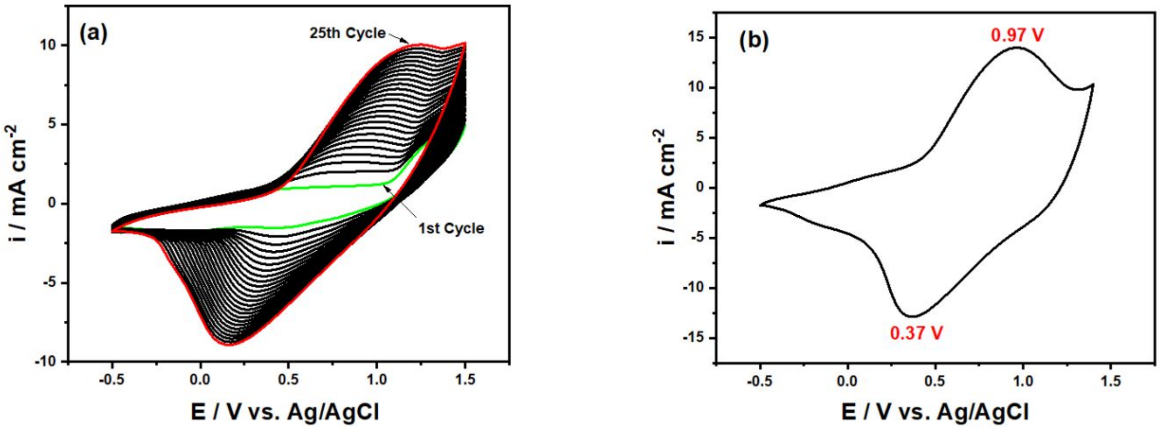

Figure S1. a) Electrochemical polymerization of DTP-Ph-Pyr monomer with a scan rate of 100 mV/s in the medium of 0.1 M $TBAPF_6$/DCM b) Electrochemical characterization of P(DTP-Ph-Pyr) polymer flm with a scan rate of 100 mV/s in the medium of 0.1 M $TBAPF_6$/ACN

Synthesis and polymerization of 4-(4H-dithieno[3,2-b:2',3'-d]pyrrol-4-yl)aniline (DTP-Ph-NH_2) monomer

By adding 2,2'-bithophene, bromine and zinc in glacial acetic acid, 3,3'-dibromo-2,2'-bitiophene synthesis was reacted. Equal amounts of 3,3'-Dibromo-2,2'-bitiophene and diamine compound, BINAP, t-BuONa and $Pd_2(dba)_3$ catalyzed coupling reaction with 4-(4H-dithieno[3,2-b:2', The 3'-d]pyrrol-4-yl)aniline (DTP-Ph-NH_2) monomer was synthesized with a 20 % yield [4].

^{1}H-NMR (400 MHz, $CDCl_3$):δ 7.37 (d, *J* = 8.6 Hz, 2H), 7,16 (d, *J* = 5.3 Hz, , 2H), 7.10 (d, *J* = 5.3 Hz, 2H), 7,84 (d, *J* = 8.6 Hz, 2H), 3.84-3.78 (bs, NH_2, 2H).

^{13}C-NMR (100 MHz, $CDCl_3$): δ 144.94, 144.61, 131.15, 124.51, 123.07, 115.92, 115.80, 112.08.

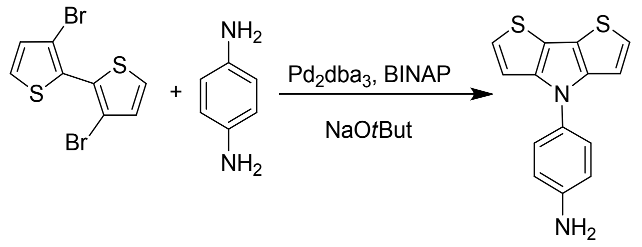

Scheme S1. Schematic representation of the synthesis of 4-(4H-dithieno[3,2-b:2',3'-d]pyrrol-4-yl)aniline (DTP-Ph-NH_2) monomer.

Homopolymerization of DTP-Ph-NH_2 monomer

DTP-Ph-NH_2 monomer (3x10^{-2} M) was electropolymerized via CV method in 0.1 M $TBAPF_6$/dichloromethane/acetonitrile medium, with a scan rate of 100 mV/s. In the presence of Ag/AgCl reference electrode, the oxidation peak of the monomer was 1.25 V. Following monomer oxidation, an electroactive polymer film developed on the gold electrode and broad oxidation and reduction peaks of the polymer film were observed in CV. With repeated transformations, increases in the current and thickness of the polymer film were obtained (Figure S2) [4].

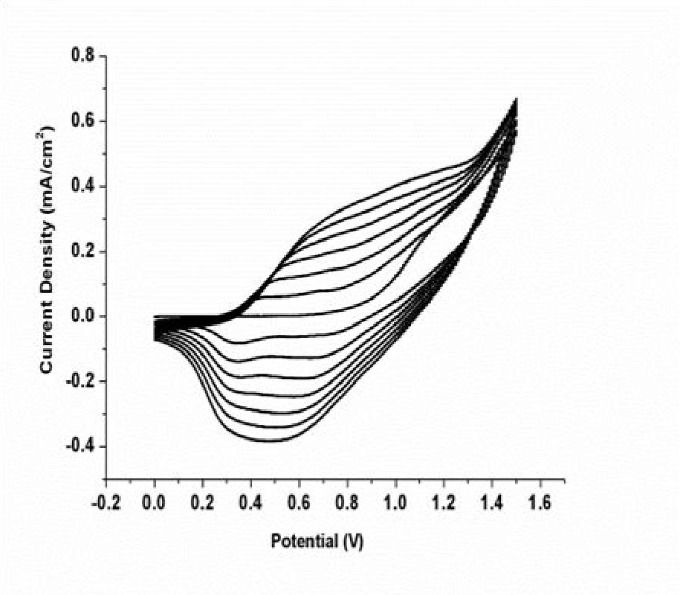

Figure S2. CV of DTP-Ph-NH_2 monomer in 0.1 M $TBAPF_6$/dichloromethane/acetonitrile medium with a scan rate of 100 mV/s in N_2 atmosphere.

Preparation of thioaniline functionalized platinum nanoparticles

A modified synthesis method for PtNPs, as reported by Perez et al. [5] was utilized. Three separate solutions were used in the synthesis of the platinum nanoparticles. The first solution was prepared by dissolving 300 mg of $PtCl_4$ in 75 mL of hexylamine. The second solution was prepared by dissolving 0.0028 moles of thioaniline and 0.011 moles of the 2-mercaptoethane sulfonic acid sodium salt in a 30 mL of 1:1 volume ratio methanol / hexane solution. Solution number three was prepared by dissolving 300 mg sodium borohydride to a 1: 1 volume of a 40 mL water/methanol mixture. Then, the solution number three was mixed vigorously with the solution number one and waited until the color of the mixture turned to brown in a few seconds, and after 1 minute, the solution number two was added to the brown mixture. After three minutes, 200 mL of distilled water was added to the mixture and stirred for 15 minutes at room temperature. The mixture was then transferred to the separatory funnel and the water phase was removed from the medium. The organic phase was washed with 200 mL of water repeatedly. The volume organic phase was then reduced to 3-4 mL with a rotary evaporation system applying 35 °C. After that, 0.0028 moles thioaniline and 0.011 moles of mercaptoethane sulfonic acid sodium salt dissolved in 15 ml of ethanol were added to the organic phase and the resulting mixture was stirred overnight at room temperature. The black precipitate was collected after several times centrifugation and each step washed with diethyl ether.

The resulting Pt nanoparticles were characterized by SEM analyzes (Figure S3).

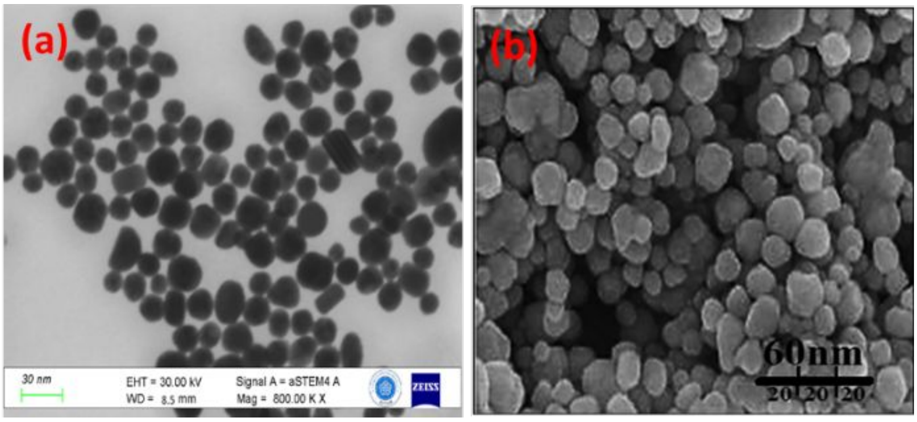

Figure S3: a) Scanning Transmission Electron Microscope (STEM) (Scale 30 nm) and b) Field Emission Scanning Electron Microscope (FESEM) (Scale 60 nm) images of thioaniline functionalized PtNPs.

Thioaniline functionalized PtNPs were subjected to electrochemical polymerization using the electropolymerization technique in a phosphate buffer solution with a pH of 7.4. Polymerization occurred within a potential range of -0.5 V to +0.5 V using cyclic voltammetry. The resulting polymer was then attached to the previously electropolymerized DTP-Ph-NH2 polymer on a gold electrode surface using the same electropolymerization method. Figure S4 illustrates the cyclic voltammogram obtained following the electrochemical attachment of PtNPs to P(DTP-Ph-NH_2). In the cyclic voltammogram, two quasi-reversible redox waves were observed. The first redox wave, centered at 0.1 V (with Ag/AgCl as the reference electrode, Eox 0.145 V vs Ag/AgCl and Ered 0.04 V vs AgCl), was attributed to the redox reaction of bisaniline cross-linking units. The second redox wave, centered at -0.30 V (Eox -0.20 V vs AgCl; Ered -0.36 V vs AgCl), was explained as the binding of Pt ions to the P(DTP-Ph-NH_2) modified surface (Figure S3). The graph in the figure displays the peak potentials corresponding to the oxidation of aniline dimer cross-linking units at different pH values [6, 7].

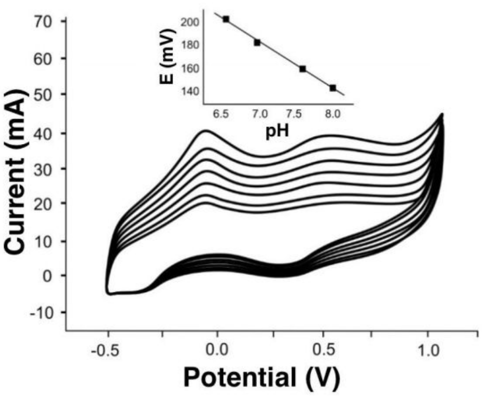

Figure S4. The cyclic voltammogram of thioaniline functionalized PtNPs attached to P(SNS-Ph-NH_2) through electrochemical polymerization.

This voltammogram of Figure S4 was obtained in an electrolyte solution containing 0.1 M phosphate buffer (pH=7.4) under argon gas, and measurements were taken at a scan rate of 100 mV/s.The DTP-Ph-NH_2 monomer underwent electropolymerization on the Au electrode surface in a medium of 0.1 M NaClO4/0.1 M LiClO4/acetonitrile solution through cyclic voltammetry. Subsequently, Pt NPs functionalized with mercaptoaniline were polymerized using the electropolymerization method in a phosphate buffer medium with pH = 7.4 via cyclic voltammetry. This process resulted in the bonding of Pt NPs to the P(DTP-Ph-NH2) polymer, which was electropolymerized on the Au electrode. The appearance of a redox pair around 0.1 V in the cyclic voltammogram signifies the formation of bis-aniline cross-links.

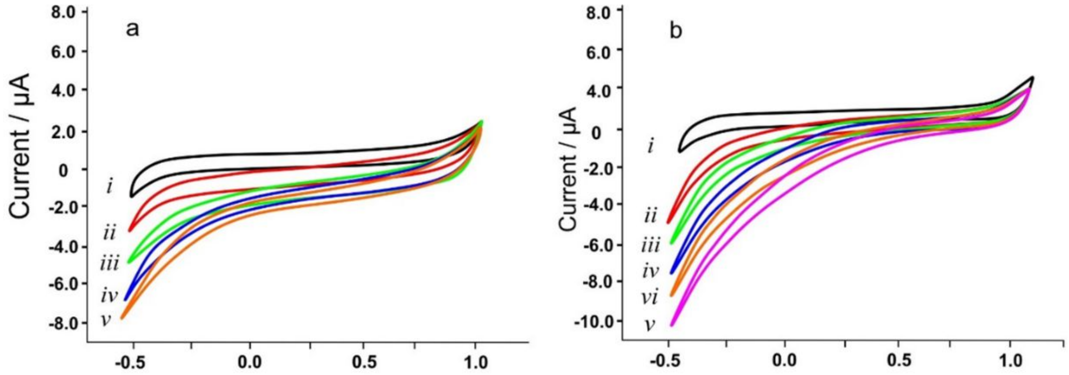

Figure S5. a) P(DTP-Ph-NH_2) coated gold electrode, i) 0, ii) 40, iii) 60, iv)100, and v) 80 cycles of P(DTP-Ph-NH_2)/Pt Nanoparticle modified gold electrodes obtained as a result of electropolymerization of Pt nanoparticles. Cyclic voltammograms in the presence of 9 mM $H_2O_{2;}$ b) P(DTP-Ph-NH_2)/Pt obtained as a result of 80 cycles electropolymerization of Pt nanoparticles on DTP-Ph-NH_2 monomer polymerized by electropolymerization with i) 0, ii) 40, iii) 60) iv) 80 v) 100 and vi) 120 cycles. Cyclic voltammograms of nanoparticle modified gold electrodes in the presence of 9 mM $H_2O_{2.}$

Table S1. Statistical data for the hydrogen generation studies

Hydrogen Generation After 30 Minutes Illumination	Data (mol/cm^3)	Standard Deviation	Error (%)
**Photosynthetic**	34.2 x 10^{-8}<br>33.2 x 10^{-8}<br>35.3 x 10^{-8}	1.05	3.07
**Respiratorial**	8.95 x 10^{-9}<br>8.50 x 10^{-9}<br>9.40 x 10^{-9}	0.45	5.0

References

[1[K. Hasan, H.B. Yildiz, E. Sperling, P.´O. Conghaile, M.A. Packer, D. Leech, C. Hagerhall, L. Gorton, Photo-electrochemical communication between cyanobacteria (Leptolyngbia sp.) and osmium redox polymer modified electrodes, Physical Chemistry Chemical Physics 16 (2014) 24676–24680, https://doi.org/10.1039/C4CP04307C.

[2] R.J. Porra, W.A. Thompson, P.E. Kriedemann, Determination of Accurate Extinction Coefficients and Simultaneous-Equations for Assaying Chlorophyll-a and Chlorophyll-B Extracted with 4 Different Solvents - Verification of the Concentration of Chlorophyll Standards by Atomic-Absorption Spectroscopy, Biochimica Biophysica Acta 975 (1989) 384-394, https://doi.org/10.1016/S0005-2728(89)80347-0.

[3] B. Bezgin Carbas, N.M Ergin, H.B. Yildiz, A. Kivrak, Electrochemical and optical properties of poly(4-(4-(1H-pyrrol-1-yl)phenyl)-4Hdithieno[3,2-b:2',3'-d]pyrrole, Polymer Bulletin 81 (2024) 9073-9089, https://doi.org/10.1007/s00289-024-05139-7.

[4] U.Y. Arslan, H.B. Yildiz, H. Azak, E. Sahin, O.Talaz, Oktay, A. Cirpan, L. Toppare, Synthesis and spectroelectrochemistry of dithieno(3,2-b:2′,3′-d)pyrrole derivatives, Journal of. Applied Polymer Science 131 (2014) 40701, https://doi.org/10.1002/app.40701.

[5] H. Perez, J.P. Pradeau, P.A. Albouy, J. Perez-Omil, Synthesis and characterization of functionalized platinum nanoparticles, Chemistry of Materials 11 (1999) 3460-3463, https://doi.org/10.1021/cm991013i.

[6] E. Granot, F. Patolsky, I. Willner, Electrochemical Assembly of a CdS Semiconductor Nanoparticle Monolayer on Surfaces: Structural Properties and Photoelectrochemical Applications, J.Phys. Chem. B. 108 (2004) 5875-5881
[7] R. Tel-Vered, H. B. Yildiz, Y. M. Yan, I. Willner, Plugging into Enzymes with Light: Photonic ‘‘Wiring’’ of Enzymes with Electrodes for Photobiofuel Cells, Small 6 (2010) 1593-1597, https://doi.org/10.1002/smll.201000296.
